# Controlling biodiversity impacts of future global hydropower reservoirs by strategic site selection

**DOI:** 10.1038/s41598-020-78444-6

**Published:** 2020-12-11

**Authors:** Martin Dorber, Anders Arvesen, David Gernaat, Francesca Verones

**Affiliations:** 1 Department of Energy and Process Engineering, NTNU, Høgskoleringen 5, 7491 Trondheim, Norway; 2 PBL - Netherlands Environment Assessment Agency, The Hague, The Netherlands; 3 Copernicus Institute of Sustainable Development, Utrecht University, Utrecht, The Netherlands

**Keywords:** Ecological modelling, Environmental impact, Hydroelectricity

## Abstract

Further reservoir-based hydropower development can contribute to the United Nations’ sustainable development goals (SDGs) on affordable and clean energy, and climate action. However, hydropower reservoir operation can lead to biodiversity impacts, thus interfering with the SDGs on clean water and life on land. We combine a high-resolution, location-specific, technical assessment with newly developed life cycle impact assessment models, to assess potential biodiversity impacts of possible future hydropower reservoirs, resulting from land occupation, water consumption and methane emissions. We show that careful selection of hydropower reservoirs has a large potential to limit biodiversity impacts, as for example, 0.3% of the global hydropower potential accounts for 25% of the terrestrial biodiversity impact. Local variations, e.g. species richness, are the dominant explanatory factors of the variance in the quantified biodiversity impact and not the mere amount of water consumed, or land occupied per kWh. The biodiversity impacts are mainly caused by land occupation and water consumption, with methane emissions being much less important. Further, we indicate a trade-off risk between terrestrial and aquatic biodiversity impacts, as due to the weak correlation between terrestrial and aquatic impacts, reservoirs with small aquatic biodiversity impacts tend to have larger terrestrial impacts and vice versa.

## Introduction

A substantial increase in the share of renewable energy in the global energy mix is needed if we are to secure a sustainable world^1^. Reservoir-based hydropower can play an important role in future energy supply, because it can provide affordable, flexible and reliable electricity with a comparably low carbon footprint^2^. Further hydropower development can contribute to fulfill the United Nations’ sustainable development goal (SDG) 7 (Affordable and clean energy) and SDG 13 (Climate action)^3^. In addition, the IPCC highlights that in all assessed mitigation pathways limiting global warming to 1.5 °C (with no or limited overshoot), up to 85% of the total electricity demand has to be produced from renewable energy sources^4^.

A study by Gernaat et al.^5^ identified globally 1,956 possible new hydropower reservoirs with a remaining economic hydropower potential of 3.9 PWh yr^−1^ (Supplementary Information (SI), section S1). “Remaining” describes that establishing these reservoirs would not interfere with nature protection areas or existing hydropower installations and “economic” refers to an electricity production cost below 0.1 US$ per kWh (henceforward we will refer to the remaining economic hydropower potential as hydropower potential). Thus, construction of these reservoirs could almost double the current global hydropower production of 4.3 PWh yr^−1^ (2019)^6^.

Notwithstanding the climate and other benefits of hydropower development^2,7–11^, reservoirs cause ecological impacts^12,13^ due to land flooding, freshwater habitat alteration, water quality degradation, and greenhouse gas emissions (GHG)^14^. The construction of all the 1,956 identified reservoirs from Gernaat et al.^5^ would imply a new global reservoir area of 240,000 km^2^, which would represent almost a doubling of the actual global reservoir area (300,000 km^2^)^15^.

This flooding of land leads to a habitat loss for terrestrial species^16^ for as long as the reservoir exists. Simultaneously, this inundation creates a large open water surface, from which permanent evaporation will occur during ice free periods^17^, in contrast to the previous terrestrial area. This increase in evaporation^17^ reduces the yearly average discharge downstream of the hydropower reservoir^18^, which in turn leads to disturbances in freshwater habitats^19^, amongst other impacts^20^. Finally, reservoirs can lead to increased anaerobic decomposition of organic matter, and thus increased GHG emissions in the form of methane^15^. These emissions contribute to climate change and the related temperature increase can accelerate the extinction of species^21^. Because of all these potential impacts, future hydropower development may interfere with SDG 6 (Clean water and sanitation) and SDG 15 (Life on land)^22^, as it may contribute to local species extinctions^23^, of fish and macroinvertebrate species^19,24^, as well as terrestrial flora and fauna^25,26^. As human-well-being ultimately relies on biodiversity and their ecosystem services^27^, conservation of biodiversity has been identified as a key parameter for sustainable development^3,28–31^. However, as Winemiller et al.^32^ pointed out, many new hydropower projects underestimate their related impact on biodiversity. To avoid the arising trade-off between SDGs 7 and 13 on the one hand and SDGs 6 and 15 on the other hand^33,34^, a quantitative assessment of potential biodiversity impacts from new hydropower projects is needed^35^. While many recent regional (e.g. refs.^8,36–39^) and global studies (e.g. refs.^2,11,18,40–43^) have assessed how hydropower electricity production can affect the state of the environment, only a few studies have quantified the resulting biodiversity impacts on a global scale (e.g. refs.^44–47^). The mentioned global biodiversity impacts assessments are however either limited by species coverage or by the spatial detail included.

In this study, we present the first global and spatially explicit assessment of terrestrial and aquatic biodiversity impacts of possible future hydropower reservoirs. We combine a high-resolution, location-specific, technical assessment from Gernaat et al.^5^ with newly developed life cycle impact assessment models for terrestrial and freshwater biodiversity to answer the following questions: Where can we produce 1 kWh hydropower with the least biodiversity impact? How much biodiversity impact can be avoided by not exploiting the full hydropower potential? How much does site selection require a trade-off between terrestrial and aquatic biodiversity impacts?

We collected site-specific environmental information from geographic information system (GIS**)** databases^48,49^ for all the possible future reservoirs from Gernaat et al.^5^. Next, we quantified reservoir-specific net land occupation [m^2^*yr]^16^, net water consumption [m^3^]^50^ and methane emissions [kg CO2 eq.]^41^ per unit of electricity produced. Finally, we quantified the terrestrial and aquatic biodiversity impact in units of potentially disappeared fraction of species (PDF), taking local species richness and environmental conditions into account^51^.

## Results

### Terrestrial biodiversity impact

**Figure 1:**
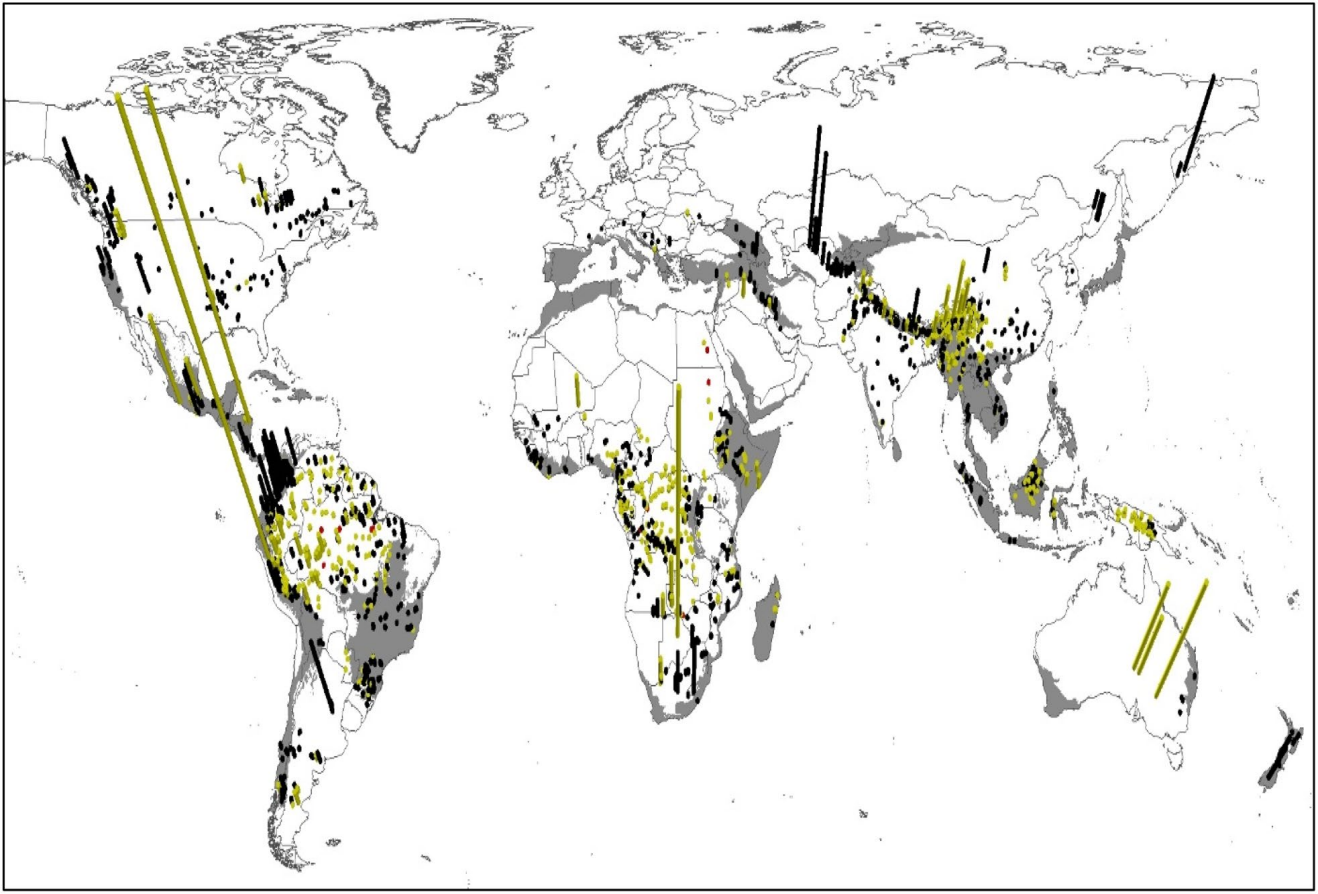
Bars show the summed terrestrial biodiversity impact of land occupation and methane emissions in PDF/kWh per reservoir. The scale of the vertical y-axis goes from 3.0 × 10^−18^ to 4.4 × 10^−12^ PDF/kWh. The color coding categorizes the yearly electricity production of each reservoir: Red =  > 30,000 GWh/y; yellow = 1000 GWh/y–30,000 GWh/y; black =  < 1000 GWh/y. Grey areas represent the biodiversity hotspots^53^ of the world^92^. Created with ArcMap 10.8 (ref.^93^).

We quantified a terrestrial biodiversity impact per kWh for 1938 future hydropower reservoirs (sum of land occupation and methane emissions impacts), between 4.4 × 10^−12^ PDF*y/kWh and 3.0 × 10^−18^ PDF*y/kWh (Fig. 1). This implies that the reservoir with highest terrestrial biodiversity impact produces one kWh with 1,475,000 times the impact of the reservoir with lowest terrestrial biodiversity impact. Globally, 10% (194) of the possible future hydropower reservoirs produce electricity with terrestrial impacts below 1.1 × 10^−15^ PDF*y/kWh, 50% (970) of the reservoirs have impacts below 8.3 × 10^−15^ PDF*y/kWh, and 10% (194) are above impacts of 6.5 × 10^−14^ PDF*y/kWh. We did not find a strong correlation between PDF*y/kWh and US$ per kWh (R^2^ of 0.008), reservoir surface area (R^2^ of 0.06) or methane emissions per kWh (R^2^ of 0.07).

Neither are the reservoirs with high or low impacts located in one specific region of the world (Fig. 1). The three most important regions in terms of cumulative electricity generation potential are Asia, South America and Africa and reservoirs with comparably low and high impact can be found distributed across all of them. In Asia, for example, the terrestrial biodiversity impact varies between 6.5 × 10^−17^ PDF*y/kWh and 4.4 × 10^−18^ PDF*y/kWh.

The terrestrial biodiversity impact from methane emissions (varying globally between 2.8 × 10^−19^ PDF*y/kWh and 5.7 × 10^−13^ PDF*y/kWh), only has a median contribution of 3% (varying between 0,01% and 86%) to the total terrestrial biodiversity impact. The variation in the total terrestrial biodiversity impact is mainly related to the land occupation biodiversity impact (varying between 2.4 × 10^−18^ PDF*y/kWh and 4.4 × 10^−12^ PDF*y/kWh; SI, S5).

The variation of the methane emission can be explained by differences in area to electricity production ratio, and maximum air temperature. The higher the air temperature and the larger the area to electricity production ratio, the higher the methane emissions and the associated terrestrial biodiversity impact. With a R^2^ of 0.91 the area to electricity production ratio was the main explanatory factor of the methane emissions (average of 3.38 g/kWh; SI, S4). Interestingly, reservoirs in the tropics (average of 1.1 × 10^−15^ PDF*y/kWh) did not have a higher per kWh methane emission biodiversity impact than reservoirs located in non-tropic regions (average of 2.4 × 10^−15^ PDF*y/kWh). However, the 20 reservoirs with the lowest methane emission impact are all located in non-tropic regions.

The biodiversity impact of land occupation depends on the m^2^*y/kWh land occupation (SI, S2) and the PDF per m^2^of land occupied. In practice, a low m^2^*y/kWh value is either the result of an already existing large water surface area prior to inundation, or a steep slope of the region in which the hydropower reservoir is sited, or the combination of both. The lowest land occupation value of 6.7 × 10^−05^ m^2^*y/kWh, for the reservoir located close to Paducah in the USA, can be explained with the fact that 60% of the water area of the reservoir already existed prior to inundation and reservoir creation. Further, reservoirs in regions with steep slopes tend to get a comparably low land occupation value because they can use a high hydropower head for electricity production while inundating a smaller area to store the same water volume as reservoirs in regions with flat slopes. This explains why the reservoir with the second lowest land occupation value of 2.7 × 10^−04^ m^2^*y/kWh is located in the Sierra de San Marcos mountain range in Mexico. In contrast, the reservoir with the highest land occupation value of 9.5 m^2^*y/kWh, is located between the Munga-Thirri-Simpson and the Tirari Desert in Australia, a flat area with no standing water area prior to inundation. The PDF per m^2^ land occupation depends on the inundated land cover type, terrestrial ecoregion area, species richness and global extinction probability (GEP). Inundating natural habitat in a small ecoregion, with high species richness and high related GEP thereby results in a high PDF per m^2^ land occupation value.

The overall low R2 of 0.06 between the land occupation itself and the biodiversity impact from land occupation shows that the land occupation of the hydropower reservoirs itself explains very little of the variance in the results. It is the PDF per m^2^ and consequently the local environmental variations (inundated land cover type, terrestrial ecoregion area, species richness and global extinction probability) that are the dominant explanatory factors of the variance in the quantified terrestrial biodiversity impact and not the pure amount of land occupied per kWh. For example, the possible future hydropower reservoir located in the Baxoi county region in Tibet has with 0.15 m^2^*y/kWh an approximately twice as big land occupation value as the reservoir located next to Firozpur in India (0.06 m^2^*y/kWh). However, as the reservoir in Tibet is located in the “The Northwestern Thorn Scrub Forests” ecoregion, which is approximately 6 times larger than the *“*Nujiang Langcang Gorge alpine conifer and mixed forests” ecoregion, it gets with 1.5 × 10^−14^ PDF*y/kWh, an approximately 13 times lower land occupation biodiversity impact than the reservoir in India (2.0 × 10^−13^ PDF*y/kWh). However, when comparing reservoirs located in the same ecoregion it is the land occupation value that defines the biodiversity impact. For example, in Europe 17 out of 44 reservoirs are in the same ecoregion, resulting in a R^2^ of 0.49 between land occupation and the related terrestrial biodiversity impact.

**Figure 2:**
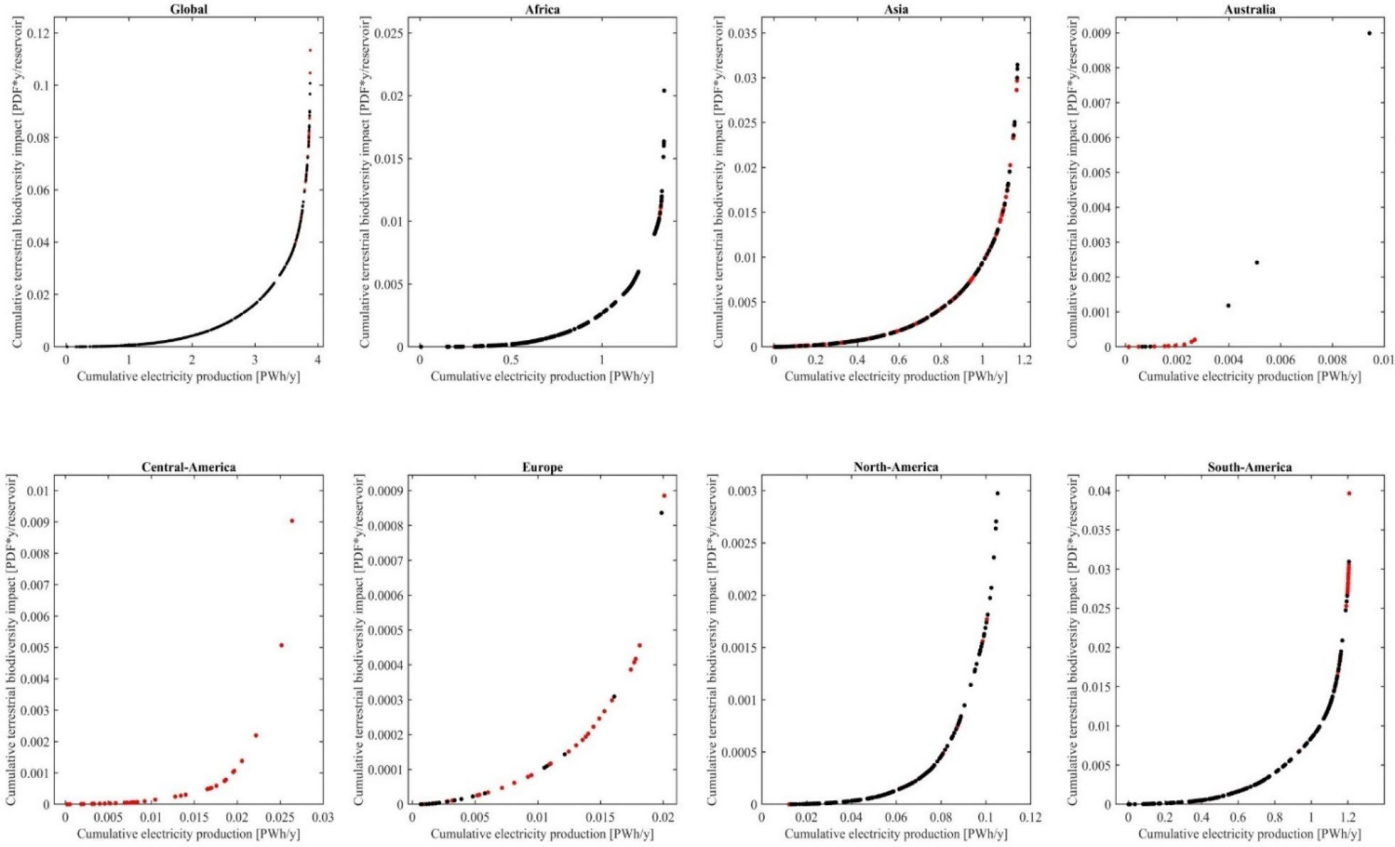
Cumulative potential terrestrial biodiversity impact of land occupation and methane emissions in PDF*y per reservoir, compared to the cumulative electricity production in PWh yr^−1^. Red points = reservoirs located in biodiversity hotspots^53^; Black points: reservoirs not located in biodiversity hotspots^53^.

Furthermore, 906 of the possible future hydropower reservoirs are located in so called biodiversity hotspots^52,53^, which are characterized as areas with high endemic species richness and where biodiversity is already threatened (Figs. 1 and 2). Partially these aspects are considered in our analysis by using the GEP, as it considers endemism and threat status of species. This can explain why hydropower reservoirs situated in biodiversity hotspots have on average a 30% higher terrestrial biodiversity impact per kWh (average of 4.28 × 10^−14^ PDF*y/kWh) than reservoirs outside biodiversity hotspots (average of 3.28 × 10^−14^ PDF*y/kWh).

The variation of the terrestrial biodiversity impacts between the reservoirs enables the possibility for strategic reservoir site-selection at the macro-level, which prioritizes reservoirs with low terrestrial biodiversity impact. But as not only the biodiversity impact per kWh varies, but also the annual electricity production at each reservoir (Fig. 1), we show the cumulative potential terrestrial biodiversity impact per reservoir as a function of cumulative electricity generation in Fig. 2. Possible future hydropower reservoirs located in the flat slope of the curves in Fig. 2 add relatively more electricity to the hydropower potential than to the terrestrial biodiversity impact of this region (biodiversity-impact-wise best hydropower reservoirs). For example, constructing the hydropower reservoir in the Waimakariri River, New Zealand, would add 1.4% to the cumulative electricity production of the region of Australia, while only adding 0.01% to its cumulative terrestrial biodiversity impact.

In contrast, hydropower reservoirs located in the steep slope of the curves in Fig. 2 do not add much electricity to the hydropower potential but add a comparably high amount to the terrestrial biodiversity impact of this region (biodiversity-impact-wise worst hydropower reservoirs). For example, constructing the possible future hydropower reservoirs in the Meranon river, Peru, would only add 0.2% to the cumulative electricity production of South America, while adding 22% to its cumulative terrestrial biodiversity impact.

All regions in Fig. 2 show curves with exponential slopes, highlighting that in all regions a comparably big proportion of the terrestrial biodiversity impact can be avoided by not realizing all the hydropower potential.

Globally, 3.9% of the hydropower potential accounts for 51% of terrestrial biodiversity impact. In other words, already half of the terrestrial biodiversity impact would be avoided, if only the other 96% of the global hydropower potential would be used. If the biodiversity-impact-wise best hydropower reservoirs would be built to reach 50% of the global hydropower potential (1.95 PWh yr^−1^), 97% (0.109 PDF*y) of the terrestrial biodiversity impact could be avoided. If globally 75% of the global hydropower potential (2.91 PWh yr^−1^) is reached by building the biodiversity-impact-wise best hydropower reservoirs, still 87% (0.099 PDF*y) of the terrestrial biodiversity impact could be avoided (SI, S7).The same general trend can be observed on a regional level. In Central America, for example, 84% of the hydropower potential could be used, while only causing 25% of the biodiversity impact. However, there are differences between the regions, as in Central-America 10% of the terrestrial biodiversity impact is caused when using 74% of the hydropower potential, while in Europe the same biodiversity impact is caused by using only 47% of the hydropower potential (SI, S7).

### Aquatic biodiversity impact

**Figure 3:**
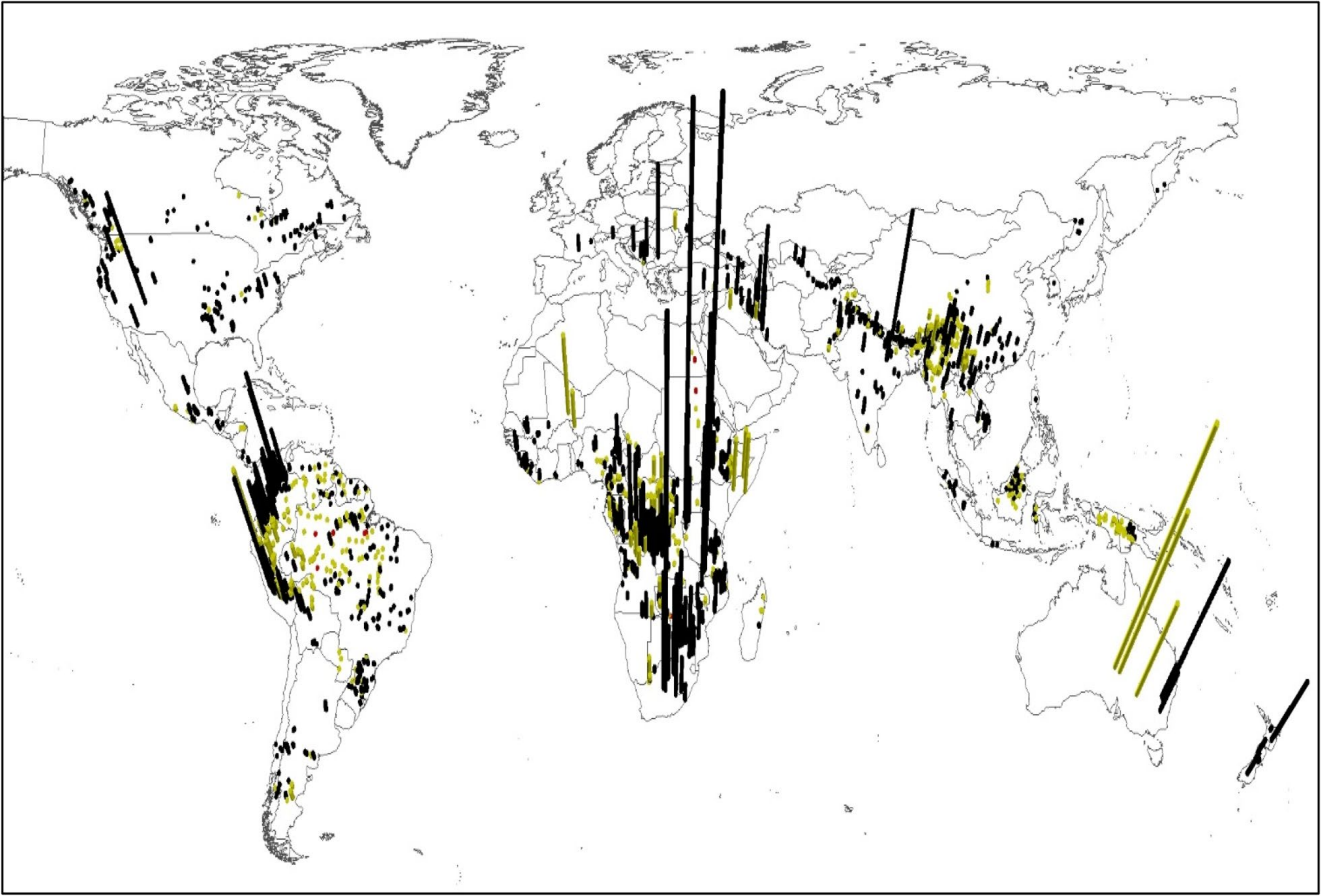
Bars show the aquatic biodiversity impact of water consumption and methane emissions in PDF/kWh per reservoir. The scale of the vertical y-axis goes from 4.7 × 10^−13^ PDF*y/kWh to 1.5 × 10^−18^ PDF*y/kWh. The color coding categorizes the yearly electricity production of each reservoir: Red =  > 30,000 GWh/y; yellow = 1000 GWh/y–30,000 GWh/y; black =  < 1000 GWh/y. World borders obtained from ref.^92^ and created with ArcMap 10.8 (ref.^93^).

The potential aquatic biodiversity impact per kWh of the 1933 possible hydropower reservoirs varies between 4.7 × 10^−13^ PDF*y/kWh and 1.5 × 10^−18^ PDF*y/kWh (Fig. 3). Globally, 10% (193) of the hydropower reservoirs produce electricity with impacts below 1.2 × 10^−16^ PDF*y/kWh, 50% (967) of the reservoirs below 1.4 × 10^−15^ PDF*y/kWh, and 10% (193) above 1.8 × 10^−14^ PDF*y/kWh. We did not find a strong correlation between PDF*y/kWh and US$ per kWh (R^2^ of 0.04), reservoir surface area (R^2^ of 0.06) and methane emissions per kWh (R^2^ of 0.12).

Figure 3 follows the same trend as Fig. 1 and shows that in all region’s hydropower reservoirs with comparably low and high biodiversity impact per kWh can be found. In Asia, for example, the aquatic biodiversity impact per kWh varies between 1.4 × 10^−13^ PDF*y/kWh and 3.0 × 10^−18^ PDF*y/kWh. The biodiversity impact of water consumption depends on the amount of water consumed to produce a kWh [m^3^/kWh] (SI, S3) and the PDF per m^3^ water consumed. The water consumption was obtained by subtracting the evaporation prior to inundation from the evaporation of the possible future reservoir.

The higher the ratio between water surface area prior to inundation and reservoir area, the smaller the difference between the two evaporations rates. A large land inundation value will therefore result in a comparably large water consumption value, confirmed by the strong correlation (R^2^ of 0.97) between amount of land occupied and amount of water consumed per kWh. The PDF per m^3^ water consumed depends on the Species-Discharge Relationship (SDR), river location, river discharge and the GEP of the freshwater groups present in the freshwater ecoregion.

Rivers with comparably low discharge tend to get comparably high regional PDFs, as they lose more species per m^3^ water consumed, and they additionally have a comparably low fish species richness. Since we used two different sets of SDRs, rivers located between 42 degree north and south, get a comparably higher PDF per m^3^ water consumed, when located in a river with the same size, than rivers in the higher latitudes.

However, the low R^2^ (0.09) between amount of water consumed and water consumption biodiversity impact per reservoir shows that the hydropower reservoir with the highest water consumption value does not automatically have the highest aquatic biodiversity impact. Hence, it is the PDF per m^3^ and consequently the environmental variations (river size, river location, species richness and GEP) that are the dominant explanatory factors for the variance in the quantified biodiversity impact.

**Figure 4:**
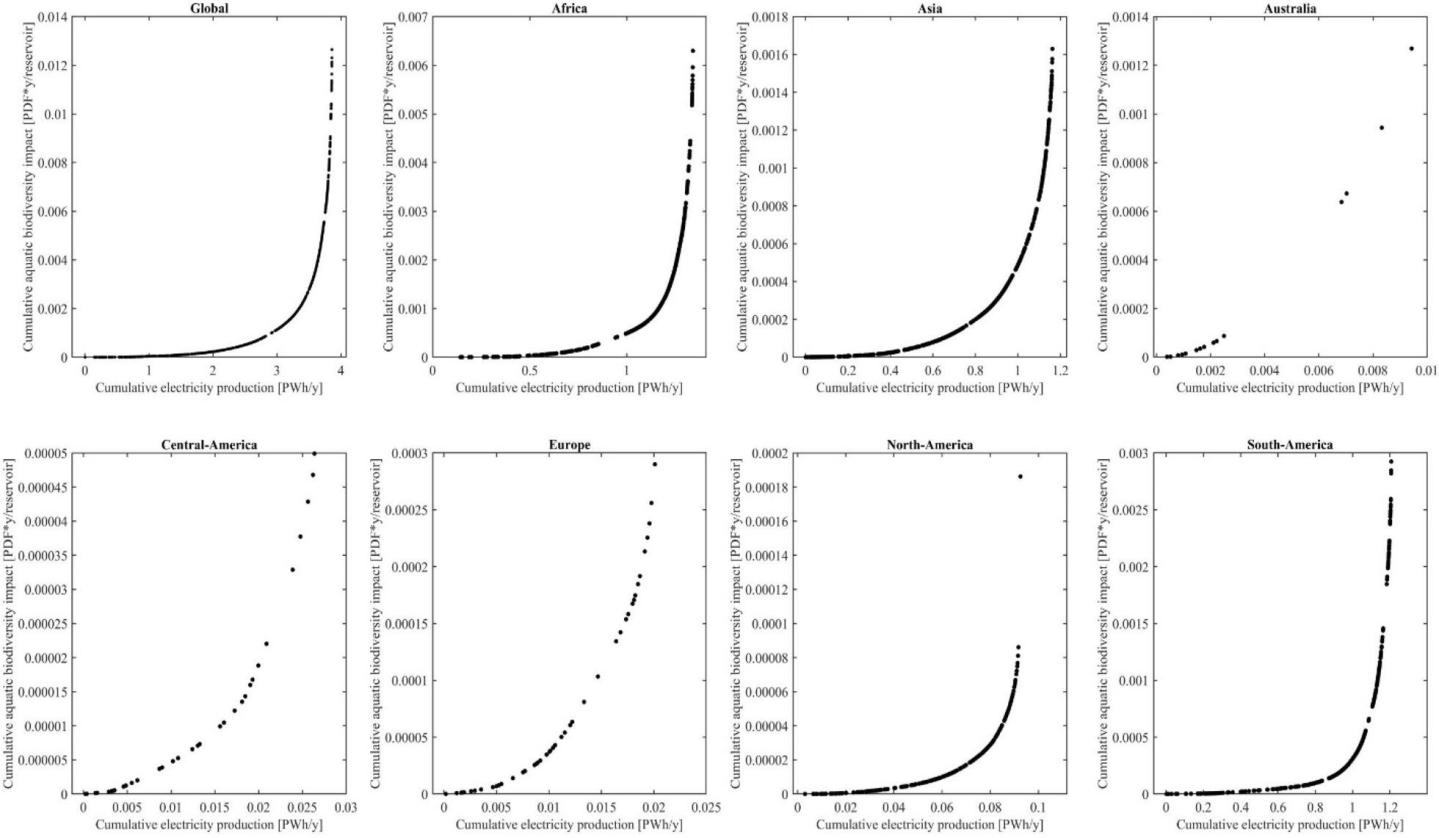
Cumulative aquatic biodiversity impact of water consumption and methane emissions in PDF*y per reservoir, compared to the cumulative electricity production in PWh yr^−1^.

When considering the different yearly electricity production of each reservoir (Fig. 3), the variation of aquatic biodiversity impacts enables the possibility for strategic reservoir site-selection at the macro-level, in order to prioritize reservoirs with low aquatic biodiversity impact. The cumulative potential aquatic biodiversity impact as a function of cumulative electricity generation is shown in Fig. 4. Here again, all regions show curves with exponential slopes, meaning that in all regions a comparably big proportion of the aquatic biodiversity impact can be avoided by not realizing a small proportion of the hydropower potential.

Globally, 2% of the hydropower potential accounts for 50% of the aquatic biodiversity impact. In other words, already half of the aquatic biodiversity impact would be avoided, if only 98% of the global hydropower potential would be used. If the biodiversity-impact-wise best hydropower reservoirs are built to reach 50% of the global hydropower potential (1.93 PWh yr^−1^), 98% (0.0124 PDF*y) of the aquatic biodiversity impact could be avoided. If globally 75% of the global hydropower potential (2.91 PWh yr^−1^) is reached by building the biodiversity-impact-wise best hydropower reservoirs, 92% (0.0117 PDF*y) of the aquatic biodiversity impact could be avoided (SI, S8). The same general trend can be observed on a regional level, as for example, in Africa 85% of the hydropower potential could be used, while only causing 14% of the region’s total biodiversity impact. However, there are difference between the regions, as in Europe 10% of the aquatic biodiversity is caused by using 82% of the hydropower potential, while in Central America the same biodiversity impact is already caused when using 39% of the hydropower potential (SI, S8).

### Overall biodiversity impact

So far, we looked at the terrestrial and aquatic biodiversity impacts individually. However, the weak correlation (R^2^ of 0.05) between the global aquatic and terrestrial biodiversity impact per kWh shows that hydropower reservoirs with low terrestrial biodiversity impact will not automatically have a low aquatic biodiversity impact too, and vice versa. As sustainable development requires hydropower reservoirs with a small terrestrial and aquatic biodiversity impact, the terrestrial and aquatic biodiversity impact should be limited to the same level (biodiversity impact limit). This can either be done by sorting (ascending order) the reservoirs by terrestrial impact and considering the parallel occurring aquatic impact (Scenario 1) as cut-off criterion, or by sorting (ascending order) the reservoirs by aquatic impacts and considering the additional terrestrial impact (Scenario 2) for cutting of at the overall biodiversity impact limit.

**Figure 5:**
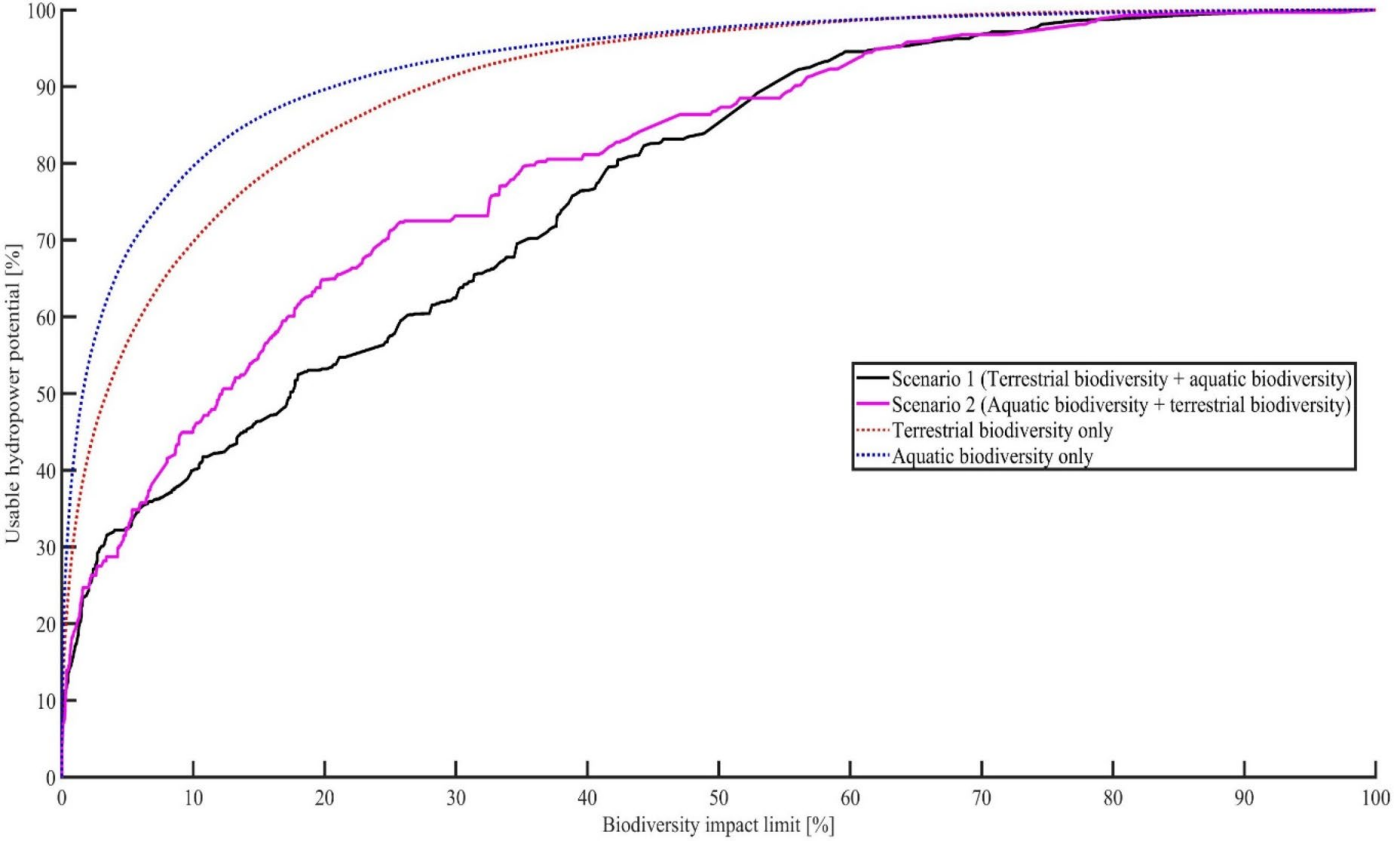
Usable hydropower potential in % (solid line) on the y-axis for Scenario 1 (black) and 2 (purple), if the biodiversity impact (terrestrial and aquatic) is limited to an equal level (x-axis), in comparison to considering biodiversity impacts (terrestrial and aquatic) individually (dotted line). Scenario 1: reservoirs sorted by terrestrial biodiversity impact and the aquatic biodiversity impacts are considered in parallel. Scenario 2: reservoirs sorted by aquatic biodiversity impact and the terrestrial biodiversity impacts are considered in parallel.

In Scenario 1 the associated aquatic biodiversity impact is larger than the terrestrial biodiversity impact, as for example 50% of the global hydropower potential is only causing 3.4% of the terrestrial biodiversity impact but 17.4% of the aquatic biodiversity impact. If the biodiversity impact limit is now set to 3.4%, only 31.6% of the hydropower potential can be used (Fig. 5). This means that in Scenario 1, the consideration of the additional aquatic biodiversity impact will lead to a reduction of the usable hydropower potential by − 18.4% (difference between dotted and solid line, Fig. 5), as opposed to only considering terrestrial biodiversity impacts. The maximum reduction in usable hydropower potential with − 32.5% is linked to a biodiversity impact limit of 16.3%.

In Scenario 2, the associated terrestrial biodiversity impact is larger than the aquatic biodiversity impact, meaning that here the consideration of the terrestrial biodiversity impact will lead to a reduction of the usable hydropower potential, in comparison to only considering aquatic biodiversity impacts (Fig. 5). The maximum reduction in usable hydropower potential in Scenario 2, with − 37% is when the biodiversity limit is 4.2%. In summary, considering aquatic and biodiversity together, will result in a more pronounced reduction of the usable hydropower potential in comparison to considering the biodiversity impacts individually.

In Scenario 2 the terrestrial impact is larger and the limiting factor, while in Scenario 1 it is the aquatic biodiversity impact. This clearly shows that the aquatic and terrestrial impacts are not correlated, and it is rather the case that reservoirs with small aquatic biodiversity impacts tend to have larger terrestrial impacts and vice versa. This highlights that there are indeed trade-offs that need to be considered between the impacts and scenarios. However, Fig. 5 also shows that the question of which scenario should be chosen to use as much hydropower potential as possible, is depending on the biodiversity impact limit. While for example, for a biodiversity impact limit of 30% it is better to choose Scenario 1, at a biodiversity impact limit of 70% Scenario 2 should be chosen. The regional results are presented in SI, S9.

## Discussion

This study illustrates how the newest set of spatially explicit life cycle impact assessment methods can be used to highlight a biodiversity trade-off for future hydropower electricity production related to the SDGs. Overall, our results show that future hydropower electricity production can have a spatially highly variable biodiversity impact, which can interfere with SDG 6 (Clean water and sanitation) and SDG 15 (Life on land), while contributing to reaching SDG 7 (Affordable and clean energy) and SDG 13 (Climate action)^9^.

We did not find a strong correlation between PDF*y/kWh and methane emissions per kWh. This in turn means, that, if mitigating climate change is the main motivation for increased hydropower production, it is likely that a potential biodiversity impact is overlooked, in spite of conservation of biodiversity having been identified as a key parameter for sustainable development^3,28–31^. This reinforces previous findings that the SDGs can be viewed as a network^4,54^ with interdependent goals^33^, and that fulfilment of one specific SDG can result in negative trade-offs with other SDGs^9^. However, our results show that careful selection of future hydropower reservoirs has a large potential to limit biodiversity impacts and can in turn help to achieve a more sustainable renewable energy development, as for example, globally 0.3% of the hydropower potential account for 25% of the terrestrial biodiversity impact.

This study is a step forward towards sustainable hydropower development, because our results show that it is the PDF/unit resource use (m^2^ or m^3^) and consequently the environmental factors (river size, ecoregion area, river location, species richness and global extinction probability) that are the dominant explanatory factors of the variance in the quantified aquatic and terrestrial biodiversity impact and not the pure amount of water consumed or land occupied per kWh. This confirms that for sustainable hydropower development, the environmental impacts have to be assessed on an impact level^47^, and should not be quantified on mere amounts of resources used [m^2^*y/kWh or m^3^/kWh]^2,43^.

As the species richness can vary significantly between different regions, our results further highlight that it is important to move from global and generic assessments to spatially explicit regional assessments^47,55^. In addition, we showed that there is a trade-off risk between terrestrial and aquatic biodiversity impacts, as construction of reservoirs with low terrestrial impacts will not automatically be accompanied by a low aquatic biodiversity impact, and vice versa. As sustainable development requires an identification of future hydropower reservoirs with the lowest possible biodiversity impact^31^, our results highlight, that it is necessary to consider aquatic and terrestrial species simultaneously and not independently^46,56^. However, there needs to be a debate for how much general biodiversity loss can be accepted. This is not (yet) answered^57^and will ultimately also involve substantial value choices by decision-makers. In any case, our study confirms that 906 of the potential hydropower reservoirs could add additional stress to already threatened terrestrial species^46^, as they are located in biodiversity hotspots. We would also like to remind the reader that hydropower is only one of several stressors for terrestrial and aquatic biodiversity loss^58,59^. Newbold et al.^60^ highlighted that approx. 60% of the world's land surface, and 9 out of 14 of the world's terrestrial biomes, have already fallen below a safe planetary boundary threshold. To fully understand how much of the hydropower potential could be used without substantially damaging the surrounding biodiversity, other biodiversity impact pathways such as impacts of discharge regulation^19^, habitat fragmentation^40^ or water regulation in the reservoir^61^ should be considered as well.

In addition, the currently developed aquatic biodiversity impact model uses only fish as biodiversity indicator, while the terrestrial biodiversity impact is based on four taxonomic groups. Inclusion of additional taxa, for example macro-invertebrates, could therefore potentially enhance the accuracy of aquatic biodiversity impact assessment^62,63^. For this, additional life cycle impact assessment model development, but also new regionalized data, is necessary^14^.

As all assessed hydropower reservoirs are economically feasible^5^, our results can support strategic decision-making at the macro-level. The applied life cycle impact assessment methodology thereby allows for a relative comparison between reservoirs^64^, to help establish a ranking of globally preferable and globally undesirable hydropower reservoirs. However, we emphasize that our results do not allow an absolute comparison of impacts^65^ and are therefore not replacing local impact assessments.

However, when using our results for developing final strategies, the limitations and uncertainties of the applied models, which will be discussed in following section, have to be considered^51^. Main contributors to uncertainty of the calculated impacts, result from uncertainties in the calculated amounts of resources used (m^3^/kwh and kg CO_2_ eq./kWh). Dorber et al.^50^ reported that, due to uncertainties of the actual evapotranspiration data^48^, the calculated water consumption could be 42.6% larger or lower. Further, the water consumption values are related to the maximum reservoir surface area. However, due to reservoir water level regulations (depending on the operation scheme of the reservoir) the reservoir might not always be completely filled^66^, leading to a temporarily reduced water surface area. In these cases, our calculated water consumption may represent an overestimation, leading to an overestimation of the total impact. As the operation scheme was not known for the possible future reservoirs^5^, the uncertainty of this temporal aspect could not be quantified. But Dorber et al.^50^ showed that reducing the reservoir surface area by 1% will result in a reduction of the water consumption by 1%. Despite the discussed uncertainty, our calculated average water consumption of 0.1 m^3^/kWh is in the range of the global average of 0.14 m^3^/kWh reported by Scherer and Pfister^41^.

To calculate the methane emission, we assumed that the reservoir will be operating for a time span of 75 years, as the reported life span of hydropower reservoirs lies between 50–100 years^67–69^. However, methane emissions decrease over the life span. Therefore, a 25 years shorter operation of an individual hydropower reservoir, would lead to a 30% underestimation of the methane emissions and, if operated 25 years longer to an overestimation of 17%. Nonetheless, our average methane emission value of 3.38 g/kWh lies between the 2.95 g/kWh reported by Scherer and Pfister^41^ and the 3.5 g/kWh reported by Hertwich et al.^70^.

Further it needs be considered that once these reservoirs are created, they could be used for other purposes like flood protection, recreation, irrigation and drinking water supply. In these cases, the biodiversity impact should be allocated between these purposes^71^, leading to a lower biodiversity impact per kWh hydropower electricity production. If for example, the rank allocation proposed by Scherer and Pfister^42^ would be used, adding a second purpose would result in a 33% lower hydropower impact, and adding 3 additional purposes would lead to a 60% lower impact.

Additional uncertainty for water consumption impacts results from the fact that no specific SDR has been developed for rivers outside 42-degree north/south^72^ and not located in Europe^50,73^. Hence, because of a lack of spatially-explicit SDRs, we assumed that 148 reservoirs, mostly located in Canada, follow the SDR developed for Central Europe^73^. However, if the SDRs in these regions would follow a SDR of a region like Norway (region 3)^50^, the water consumption impact in these regions could be 47% lower. To resolve this uncertainty, improvements in the global coverage of life cycle impact assessments models are still needed, which was beyond the scope of this study.

Furthermore, for a development of more elaborated policy-strategies, a complete life cycle assessment, including more life cycle stages, like for example dam construction and deconstruction is needed^64^ as these processes would add further impacts, leading amongst others also to a higher biodiversity impact. However, recent life cycle assessment studies indicate that the additional impacts, would vary by region. For China it has been reported that the operation phase of the reservoir accounted for 99% of the water consumption and 87% of the GHG emissions, when neglecting the deconstruction phase^74^. In addition, for the tropics it has been reported that the operation is the main contributor to GHG emissions. Hence in such regions, water consumption and GHG emission for dam construction are minor in comparison to the operation phase, meaning that the major impacts are covered in our study. However, for Norway, Bakken et al.^75^ reported that the water consumption during operation was only responsible for up to 68% of life cycle water consumption and for temperate and boreal region it has been reported that the deconstruction is the main contributor to GHG emissions^76^, while for Myanmar it has been reported that the construction phase was with up to 60% the main contributor to global warming impacts^77^. Consequently, inclusion of addition life stages in such areas could lead to an increased biodiversity impact of up to 40%, bearing in mind that due to global value changes these impacts may not necessarily occur in the same region as the reservoir location. Furthermore, a holistic assessment may include infrastructure-related land occupation impacts (construction of access roads, tunnels and power transmissions lines)^14^, as Gernaat et al.^5^ indicated that infrastructure development will mostly occur in developing regions, leading to a higher biodiversity impact in these regions.

We showed that biodiversity impacts need to be included in the final decision process, to ensure that a potential biodiversity tradeoff is not overlooked. However, biodiversity trade-offs are only one important layer of the final decision on which possible future hydropower reservoirs should be constructed, as other factors like electricity demand, social aspects or human health impacts can play additional important roles. For example, Moran et al.^78^ has pointed out that social, behavioral, cultural, economic, and political disruption that humans near dam’s face are routinely underestimated.

Finally, our results indicate that hydropower might not be the optimal renewable energy technologies to fulfil SDG 7 with minimal biodiversity trade-offs in all regions. This confirms that the fulfilment of SDG 7 most likely relies on the correct mix of renewable energy technologies^79^ and not one single type of renewable energy^1^. Therefore, policy strategies should cross-compare the biodiversity impacts of all potential renewable energy sources and should not only focus on hydropower^80^. Popescu et al.^56^ for example highlighted that hydropower performed best when constructed in montane streams, while wind power performed best in coastal areas. Such a comparison could also be done within the field of life cycle assessment. However, for wind power, a comparative set of explicit life impact assessment does not exist^81^.

The results of this study contribute to the understanding of biodiversity trade-offs associated with hydropower at an economy-wide and regional or global scale. This knowledge is important to understand the ability of hydropower to help our societies achieve both climate and biodiversity related SGDs simultaneously. Therefore, our results provide valuable information for understanding the role of hydropower in a future sustainable world, which is useful for developing strategies at the macro-level.

## Methods

### Land occupation

We adopted the method from Dorber et al.^16^ to calculate the net land occupation values [m^2^*yr/kWh] for every possible future reservoir *x* with Eq. (1). The “net” approach is accounting for the conditions prior to dam construction^82^.

1 $${\text{LO}}_{{\text{x}}} = \frac{{\mathop \sum \nolimits_{{{\text{n}} = 1}}^{{\text{k}}} {\text{LO}}_{{{\text{i}},{\text{x}}}} }}{{{\text{E}}_{x} }}$$

where E is the electricity produced with reservoir *x* in kWh per year obtained from Gernaat et al.^5^, LO_i,x_ is the land occupation of terrestrial land use type *i* (natural habitat, managed forest, agricultural area, pasture or urban area) inside reservoir *x* in m^2^ and *k* is the number of land use types inside reservoir *x*. The term $\sum_{n=1}^{k}{\mathrm{LO}}_{i}$ thereby represents the total inundated land area.

To calculate ${\mathrm{LO}}_{\mathrm{i},\mathrm{x}}$ [m^2^*y] we used the LUCM map from Dorber et al.^83^, as it ensures compatibly with the applied land occupation characterization factors (CFs). This map provides land use information for six land use types: natural habitat, managed forest, agricultural area, pasture, urban area and water at 10′′ resolution for 781 terrestrial ecoregions. We assumed that the future reservoir will be a circle around the center coordinates^5^ and calculated ${\text{LO}}_{{{\text{i}},{\text{x}}}}$ with Eq. (2).

2 $${\text{LO}}_{{{\text{i}},{\text{x}}}} = { }\frac{{{\text{Number}}\;{\text{ of}}\;{\text{ pixels}}_{{{\text{LUCM}},{\text{i}},{\text{x}}}} { }}}{{\mathop \sum \nolimits_{{{\text{i}} = 1}}^{6} {\text{Number}}\;{\text{ of}}\;{\text{ pixels}}_{{{\text{LUCM}},{\text{i}},{\text{x}}}} { }}} \times {\text{FRA}}$$

where FRA [m^2^] is the future reservoir area obtained from Gernaat et al.^5^.

### Biodiversity impacts of land occupation

The terrestrial biodiversity damage of possible future land occupation of reservoir *x* can be quantified with a characterization factor, denoting the PDF per m^2^ future land occupation. We used the global taxon aggregated CFs, from Dorber et al.^83^ quantifying the global average PDF of four taxonomic groups (mammals, birds, reptiles, amphibians) per m^2^ in one terrestrial ecoregion^84^. The CFs are specific for each terrestrial ecoregion *j* and differentiate between the inundation of natural habitat, managed forest, agricultural area, pasture or urban area. The land use types *i* explain the land use types used in Eq. (1). To convert regional PDFs (indicating a fraction of potential regional species extirpations) into global PDFs (indicating a fraction of potential global species extinctions), the method from Dorber et al.^83^ uses the categorical global extinction probability (GEP) from Kuipers et al.^85^. The GEP indicates the likelihood that species of a taxonomic group get extinct globally if they become extirpated in region j. The GEPs range from 0 to 1, and are based on local species range sizes, species threat levels, and species richness^85^.

Following, the terrestrial biodiversity impact of land occupation (ILO) for reservoir *x* in PDF*y/kWh can be calculated with Eq. (3):

3 $${\text{ILO}}_{{\text{x}}} = \frac{{\sum\nolimits_{{{\text{n }} = {\text{ }}1}}^{{\text{k}}} {\left( {CF_{{{\text{global,taxon aggregated,i,j}}}} \times {\text{LO}}_{{{\text{i,x}}}} } \right)} }}{{{\text{E}}_{{\text{x}}} }}$$

where ${\text{CF}}_{\text{global, taxon aggregated,j,i}}$ [PDF/m^2^] is the global taxon aggregated CF from Dorber et al.^83^ for land use type *i* and terrestrial ecoregion *j. k* is the number of land use types inside reservoir *x*, E is the electricity produced with reservoir *x* in kWh per year obtained from Gernaat et al.^5^ and LO_i,x_ [m^2^*y] is the land occupation of terrestrial land use type *i* inside reservoir *x*. We used the center coordinates of each reservoir from Gernaat et al.^5^ to identify in which terrestrial ecoregion *j* each reservoir *x* is located.

### Water consumption

We adopted the method from Dorber et al.^50^ to calculate the net water consumption values [m^3^/kWh] for every possible future reservoir *x* with Eq. (4).

4 $${\text{Net}}\;{\text{ water}}\;{\text{ consumption}}_{{\text{ x}}} = \frac{{\frac{{\left( {{\text{ PET}}_{{\text{ x}}} - {\text{AET}}_{{\text{ x}}} } \right) \times {\text{LO}}_{{\text{x}}} }}{1000}}}{{{\text{E}}_{{\text{ x}}} }}$$

where LO_x_ [m^2^] is the net land occupation value of reservoir *x* obtained in Eq. (1), E [kWh per year] is the electricity produced with reservoir x obtained from Gernaat et al.^5^, PET [mm/year] is the average yearly potential evapotranspiration of reservoir *x* and AET [mm/year] is the average actual evapotranspiration of reservoir *x*.

We followed Dorber et al.^50^ and obtained average yearly potential evapotranspiration and average yearly actual evapotranspiration from the MODIS Global Evapotranspiration Project (MOD16)^48,86,87^. To calculate PET and AET we assumed that the future reservoir will be a circle around the center coordinates^5^ and averaged the MOD16 PET and AET values inside reservoir *x*.

### Biodiversity impact of water consumption

To quantify the potential aquatic biodiversity damage of water consumption from reservoir *x* we used the Species-discharge relationship (SDR)^88^. The SDR relates river discharge to species richness and is the state of the art concept within the life cycle impact assessment framework for the derivation of reservoir *x* specific water consumption CFs^72,73^.

We calculated the characterization factor [PDF*y/m^3^], quantifying the impact of water consumption at the outlet of reservoir *x* on freshwater fish species with Eq. (5):

5 $${\text{CF}}_{{{\text{wc}},{\text{x}}}} = \frac{{\frac{{{\text{dS}}_{{\text{x}}} }}{{{\text{R}}_{{\text{x}}} }}}}{{{\text{dQ}}}} \times {\text{GEP}}_{{{\text{j}},{\text{g}}}} = \frac{{\frac{{\left( {{\text{b}} \times {\text{a}}} \right) \times {\text{Q}}_{{\text{x}}}^{{\left( {{\text{b}} - 1} \right)}} }}{{{\text{a }} \times {\text{Q}}_{{\text{x}}}^{{\text{b}}} }}}}{{{\text{dQ}}}} \times {\text{ GEP}}_{{{\text{j}},{\text{g}}}} { }$$

where dS is the derivative of the SDR power function used to find the fish species loss per unit change of discharge. *R* is the number of fish species predicted by the SDR. *a* and *b* are model coefficients of the SDR power function. GEP_j,g_ is the global extinction probability for freshwater groups of the freshwater ecoregion *j*^89^ in which the hydropower reservoir is located. We used the center coordinates of each reservoir from Gernaat et al.^5^ to identify in which freshwater ecoregion *j*^89^ each reservoir *x* is located. dQ is the marginal change in discharge [m^3^/y] and is in our cases always 1 m^3^/y, to link it with the net water consumption calculated in Eq. (4). Q_*x*_ [m^3^/s] is the average annual discharge at the outlet of reservoir *x.*

This CF assumes that one unit change in water consumption (e.g. 1 m^3^ evaporation) leads to one unit reduction of river discharge. For hydropower reservoirs located between 42 degree north and south we used the SDR power function from Hanafiah et al.^72^ ( R = 4.2 × Q_x_^0.4^), and for all other reservoirs the SDR power function from Tendall et al.^73^ (R = 7.82 × Q_x_^0.3^). We obtained Q_x_ from Gernaat et al.^5^. For GEP_j,g_ we used the categorical extinction probability for freshwater groups from Kuipers et al.^85^.

### Methane emissions

We used the statistical model from Scherer and Pfister^41^ to predict the biogenic methane (CH_4_) emissions [kg CH_4_ /kWh] (Eq. 6) for each reservoir *x*. We calculated the average yearly CH_4_ emissions over the reservoirs life span (LS).

6 $${\text{CH}}_{{4,{\text{x}}}} = \frac{{\mathop \sum \nolimits_{{{\text{Age}} = 1}}^{{{\text{LS}}}} \frac{{{\text{exp}}(\left( {{ } - 9.81 - 0.75{ } \times {\text{ln}}\left( {{\text{Age}}} \right) + 1.18{ } \times \ln \left( {{\text{ATE}}} \right) + { }4.5{ } \times {\text{ln }}\left( {{\text{TMX}}} \right)} \right)}}{1000}}}{{{\text{LS}}}}$$

where AGE [years] is the used age of reservoir *x*, LS [years] is the expected life span of reservoir *x*, ATE is the area to electricity ratio [km^2^/GWh] and TMX [°C] is the maximum temperature.

As before, we assumed that the future reservoir will be a circle around the center coordinates^5^ and obtained TMX from the WorldClim Version 2^49^. This dataset provides the maximum temperature between 1970 and 2000 at a 30 s resolution. The reported life span of hydropower reservoirs lies between 50–100 years^67–69^. We used 75 year as LS, as it lies in the middle of the reported lifespan. ATE was calculated with the reservoir surface area and the electricity production data from Gernaat et al.^5^. To convert methane emission values into CO_2_ equivalent (CO_2_ eq.) emission values, we used a 100-year global warming potential (GWP) value of 30.11^90^.

### Biodiversity impact of methane emissions

To quantify the potential terrestrial biodiversity damage of the methane emission at reservoir *x* in PDF*y/kWh we multiplied CH_4,x_ with “Terrestrial Ecosystems extended CF for climate change” of 1.57 × 10^−14^ PDF*y/kg CO_2_ eq. from LC-Impact^91^. To quantify the potential aquatic biodiversity damage of the methane emission at reservoir *x* in PDF*y/kWh we multiplied CH_4,x_ with “Aquatic Ecosystems extended CF for climate change” of 4.87 × 10^−15^ PDF*y/kg CO_2_ eq. from LC-Impact^91^. Both CFs are calculating global PDF values and therefore do not need a GEP correction.

### Combined biodiversity impact

We calculated the combined potential terrestrial biodiversity impact [PDF*y/kWh] by adding the terrestrial biodiversity impact of methane emissions [PDF*y/kWh] and land occupation [PDF*y/kWh]. For 16 possible future hydropower reservoirs no combined potential terrestrial biodiversity could be calculated, due to missing max temperature values for the methane emission calculation.

We also calculated the combined potential aquatic biodiversity impact [PDF*y/kWh] by adding the aquatic biodiversity impact of methane emissions [PDF*y/kWh] and from water consumption [PDF*y/kWh]. For 24 possible future hydropower reservoirs no total potential freshwater biodiversity impact could be calculated, due to missing max temperature values (15 reservoirs) and due to missing evaporation values (14 reservoirs).

### Reservoir biodiversity impact

To identify which of the hydropower reservoirs are most sustainable from a biodiversity perspective, we compare the potential biodiversity impact per reservoir [PDF*y]. This is necessary, because not only the potential biodiversity impact per kwh varies but also the energy production at each reservoir. Multiplying the total potential terrestrial or aquatic impact per kWh with the yearly electricity production of each hydropower reservoir, gives the potential yearly biodiversity impact per reservoir.

## Supplementary information

Supplementary Information.

## Data Availability

The water consumption values, methane emissions values, and related biodiversity impacts for each possible future hydropower reservoir are presented in the Supplementary Information. Additional data that support the findings of this work are available from the authors upon reasonable request.

## References

[1] Bogdanov D (2019). Radical transformation pathway towards sustainable electricity via evolutionary steps. Nat. Commun..

[2] UNEP. *Green Energy Choices: The benefits, risks and trade-offs of low-carbon technologies for electricity production*. Report of the International Resource Panel (2016).

[3] United Nations. Transforming our world: The 2030 agenda for sustainable development—A/RES/70/1. (2015).

[4] Intergovernmental Panel on Climate Change. Global Warming of 1.5°C. An IPCC Special Report on the impacts of global warming of 1.5 °C above pre-industrial levels and related global greenhouse gas emission pathways, in the context of strengthening the global response to the threat of climate change, sustainable development, and efforts to eradicate poverty. (2018).

[5] Gernaat DEHJ, Bogaart PW, Vuuren DPV, Biemans H, Niessink R (2017). High-resolution assessment of global technical and economic hydropower potential. Nature Energy.

[6] IEA. *Hydropower*. (Paris, 2020).

[7] Intergovernmental Panel on Climate Change. Hydropower. In *IPCC Special Report on Renewable Energy Sources and Climate Change Mitigation* (2011).

[8] Almeida RM (2019). Reducing greenhouse gas emissions of Amazon hydropower with strategic dam planning. Nat. Commun..

[9] Fuso Nerini F (2018). Mapping synergies and trade-offs between energy and the sustainable development goals. Nat. Energy.

[10] Muller M (2019). Hydropower dams can help mitigate the global warming impact of wetlands. Nature.

[11] Pehl M (2017). Understanding future emissions from low-carbon power systems by integration of life-cycle assessment and integrated energy modelling. Nat. Energy.

[12] Wu H (2019). Effects of dam construction on biodiversity: a review. J. Clean. Prod..

[13] Turgeon K, Turpin C, Gregory-Eaves I, Lawler J (2019). Dams have varying impacts on fish communities across latitudes: a quantitative synthesis. Ecol. Lett..

[14] Gracey EO, Verones F (2016). Impacts from hydropower production on biodiversity in an LCA framework—review and recommendations. Int. J. Life Cycle Assess..

[15] Lehner B (2011). High-resolution mapping of the world's reservoirs and dams for sustainable river-flow management. Front. Ecol. Environ..

[16] Dorber M, May R, Verones F (2018). Modeling net land occupation of hydropower reservoirs in Norway for use in life cycle assessment. Environ. Sci. Technol..

[17] Strachan IB (2016). Does the creation of a boreal hydroelectric reservoir result in a net change in evaporation?. J. Hydrol..

[18] Mekonnen MM, Hoekstra AY (2012). The blue water footprint of electricity from hydropower. Hydrol. Earth Syst. Sci..

[19] Poff NL, Zimmerman JKH (2010). Ecological responses to altered flow regimes: a literature review to inform the science and management of environmental flows. Freshw. Biol..

[20] Gillespie BR, Desmet S, Kay P, Tillotson MR, Brown LE (2015). A critical analysis of regulated river ecosystem responses to managed environmental flows from reservoirs. Freshw. Biol..

[21] Urban MC (2015). Accelerating extinction risk from climate change. Science.

[22] Hermoso V, Clavero M, Green AJ (2019). Don’t let damage to wetlands cancel out the benefits of hydropower. Nature.

[23] McAllister, D. E., Craig, J. F., Davidson, N., Delany, S. & Seddon, M. Biodiversity impacts of large dams. Background Paper Nr. 1 - Prepared for IUCN/UNEP/WCD (2001).

[24] Crook DA (2015). Human effects on ecological connectivity in aquatic ecosystems: Integrating scientific approaches to support management and mitigation. Sci. Total Environ..

[25] Alho CJ (2011). Environmental effects of hydropower reservoirs on wild mammals and freshwater turtles in Amazonia: a review. Oecologia Australis.

[26] Kitzes J, Shirley R (2016). Estimating biodiversity impacts without field surveys: a case study in northern Borneo. Ambio.

[27] Mace GM, Norris K, Fitter AH (2012). Biodiversity and ecosystem services: a multilayered relationship. Trends Ecol. Evol..

[28] Secretariat of the Convention on Biological Diversity. Global Biodiversity Outlook 4. (Montreal, 2014).

[29] Bennett EM (2015). Linking biodiversity, ecosystem services, and human well-being: three challenges for designing research for sustainability. Curr. Opin. Environ. Sustain..

[30] Opoku A (2019). Biodiversity and the built environment: Implications for the sustainable development goals (SDGs). Resour. Conserv. Recycl..

[31] Blicharska M (2019). Biodiversity’s contributions to sustainable development. Nat. Sustain..

[32] Winemiller KO (2016). Balancing hydropower and biodiversity in the Amazon, Congo, and Mekong. Science.

[33] Nilsson M, Griggs D, Visbeck M (2016). Policy: map the interactions between sustainable development goals. Nature.

[34] Bhaduri A (2016). Achieving sustainable development goals from a water perspective. Front. Environ. Sci..

[35] Liu J (2018). Nexus approaches to global sustainable development. Nat. Sustain..

[36] Shin S (2020). High resolution modeling of river-floodplain-reservoir inundation dynamics in the Mekong River Basin. Water Resour. Res..

[37] Schmitt RJP, Bizzi S, Castelletti A, Kondolf GM (2018). Improved trade-offs of hydropower and sand connectivity by strategic dam planning in the Mekong. Nat. Sustain..

[38] Pokhrel Y, Shin S, Lin Z, Yamazaki D, Qi J (2018). Potential disruption of flood dynamics in the Lower Mekong River Basin due to upstream flow regulation. Sci. Rep..

[39] Ashraf FB (2018). Changes in short term river flow regulation and hydropeaking in Nordic rivers. Sci. Rep..

[40] Barbarossa V (2020). Impacts of current and future large dams on the geographic range connectivity of freshwater fish worldwide. Proc. Natl. Acad. Sci..

[41] Scherer L, Pfister S (2016). Hydropower's biogenic carbon footprint. PLoS ONE.

[42] Scherer L, Pfister S (2016). Global water footprint assessment of hydropower. Renew. Energy.

[43] Evans A, Strezov V, Evans TJ (2009). Assessment of sustainability indicators for renewable energy technologies. Renew. Sustain. Energy Rev..

[44] Laborde A, Habit E, Link O, Kemp P (2020). Strategic methodology to set priorities for sustainable hydropower development in a biodiversity hotspot. Sci. Total Environ..

[45] Haga C (2020). Scenario analysis of renewable energy-biodiversity nexuses using a forest landscape model. Front. Ecol. Evol..

[46] Zarfl C (2019). Future large hydropower dams impact global freshwater megafauna. Sci. Rep..

[47] Gibon T, Hertwich EG, Arvesen A, Singh B, Verones F (2017). Health benefits, ecological threats of low-carbon electricity. Environ. Res. Lett..

[48] Mu Q, Zhao M, Running SW (2011). Improvements to a MODIS global terrestrial evapotranspiration algorithm. Remote Sens. Environ..

[49] Fick SE, Hijmans RJ (2017). WorldClim 2: new 1-km spatial resolution climate surfaces for global land areas. Int. J. Climatol..

[50] Dorber M, Mattson KR, Sandlund OT, May R, Verones F (2019). Quantifying net water consumption of Norwegian hydropower reservoirs and related aquatic biodiversity impacts in life cycle assessment. Environ. Impact Assess. Rev..

[51] Verones F (2017). LCIA framework and cross-cutting issues guidance within the UNEP-SETAC Life Cycle Initiative. J. Clean. Prod..

[52] Myers N, Mittermeier RA, Mittermeier CG, Da Fonseca GA, Kent J (2000). Biodiversity hotspots for conservation priorities. Nature.

[53] Critical Ecosystem Partnership Fund. *Biodiversity Hotspot Shapefile.* https://www.cepf.net/our-work/biodiversity-hotspots/hotspots-defined (2016).

[54] Le Blanc D (2015). Towards integration at last? The sustainable development goals as a network of targets. Sustain. Dev..

[55] Mutel C (2019). Overview and recommendations for regionalized life cycle impact assessment. Int. J. Life Cycle Assess..

[56] Popescu VD (2020). Quantifying biodiversity trade-offs in the face of widespread renewable and unconventional energy development. Sci. Rep..

[57] Oliver TH (2016). How much biodiversity loss is too much?. Science.

[58] Pereira HM, Navarro LM, Martins IS (2012). Global biodiversity change: the bad, the good, and the unknown. Annu. Rev. Environ. Resour..

[59] Best J (2019). Anthropogenic stresses on the world’s big rivers. Nat. Geosci..

[60] Newbold T (2016). Has land use pushed terrestrial biodiversity beyond the planetary boundary? A global assessment. Science.

[61] Eloranta AP, Finstad AG, Helland IP, Ugedal O, Power M (2018). Hydropower impacts on reservoir fish populations are modified by environmental variation. Sci. Total Environ..

[62] Worrall TP (2014). The identification of hydrological indices for the characterization of macroinvertebrate community response to flow regime variability. Hydrol. Sci. J..

[63] Holt CR, Pfitzer D, Scalley C, Caldwell BA, Batzer DP (2015). Macroinvertebrate community responses to annual flow variation from river regulation: an 11-year study. River Res. Appl..

[64] International Organisation for Standardization. ISO 14044:2006 Environmental management—Life cycle assessment—Principles and framework (2006).

[65] Jolliet O (2018). Global guidance on environmental life cycle impact assessment indicators: impacts of climate change, fine particulate matter formation, water consumption and land use. Int. J. Life Cycle Assess..

[66] Hirsch PE, Schillinger S, Weigt H, Burkhardt-Holm P (2014). A hydro-economic model for water level fluctuations: combining limnology with economics for sustainable development of hydropower. PLoS ONE.

[67] Gagnon L, Bélanger C, Uchiyama Y (2002). Life-cycle assessment of electricity generation options: the status of research in year 2001. Energy Policy.

[68] George MW, Hotchkiss RH, Huffaker R (2017). Reservoir sustainability and sediment management. J. Water Resour. Plann. Manag..

[69] Yüksel I (2010). Hydropower for sustainable water and energy development. Renew. Sustain. Energy Rev..

[70] Hertwich EG (2013). Addressing biogenic greenhouse gas emissions from hydropower in LCA. Environ. Sci. Technol..

[71] Bakken TH, Modahl IS, Raadal HL, Bustos AA, Arnoy S (2016). Allocation of water consumption in multipurpose reservoirs. Water Policy.

[72] Hanafiah MM, Xenopoulos MA, Pfister S, Leuven RSEW, Huijbregts MAJ (2011). Characterization factors for water consumption and greenhouse gas emissions based on freshwater fish species extinction. Environ. Sci. Technol..

[73] Tendall DM, Hellweg S, Pfister S, Huijbregts MAJ, Gaillard G (2014). Impacts of river water consumption on aquatic biodiversity in life cycle assessment—a proposed method, and a case study for Europe. Environ. Sci. Technol..

[74] Wang J (2019). Assessing the water and carbon footprint of hydropower stations at a national scale. Sci. Total Environ..

[75] Bakken TH, Modahl IS, Engeland K, Raadal HL, Arnøy S (2016). The life-cycle water footprint of two hydropower projects in Norway. J. Clean. Prod..

[76] Song C, Gardner KH, Klein SJW, Souza SP, Mo W (2018). Cradle-to-grave greenhouse gas emissions from dams in the United States of America. Renew. Sustain. Energy Rev..

[77] Aung TS, Fischer TB, Azmi AS (2020). Are large-scale dams environmentally detrimental? Life-cycle environmental consequences of mega-hydropower plants in Myanmar. Int. J. Life Cycle Assess..

[78] Moran EF, Lopez MC, Moore N, Müller N, Hyndman DW (2018). Sustainable hydropower in the 21st century. Proc. Natl. Acad. Sci..

[79] United Nation Environmental Program. Green energy choices: The benefits, risks, and trade-offs of low-carbon technologies for electricity production. (2016).

[80] Edenhofer, O. *et al.* IPCC special report on renewable energy sources and climate change mitigation. (Prepared By Working Group III of the Intergovernmental Panel on Climate Change, Cambridge University Press, Cambridge, UK, 2011).

[81] Laranjeiro T, May R, Verones F (2018). Impacts of onshore wind energy production on birds and bats: recommendations for future life cycle impact assessment developments. Int. J. Life Cycle Assess..

[82] Bakken TH, Killingtveit Å, Engeland K, Alfredsen K, Harby A (2013). Water consumption from hydropower plants—review of published estimates and an assessment of the concept. Hydrol. Earth Syst. Sci..

[83] Dorber M, Kuipers K, Verones F (2020). Global characterization factors for terrestrial biodiversity impacts of future land inundation in life cycle assessment. Sci. Total Environ..

[84] Olson DM (2001). Terrestrial ecoregions of the world: a new map of life on earth. Bioscience.

[85] Kuipers KJJ, Hellweg S, Verones F (2019). Potential consequences of regional species loss for global species richness: a quantitative approach for estimating global extinction probabilities. Environ. Sci. Technol..

[86] University of Montana. *MODIS Global Evapotranspiration Project (MOD16)*, http://www.ntsg.umt.edu/project/modis/mod16.php.

[87] Mu Q, Heinsch FA, Zhao M, Running SW (2007). Development of a global evapotranspiration algorithm based on MODIS and global meteorology data. Remote Sens. Environ..

[88] Xenopoulos MA, Lodge DM (2006). Going with the flow: using species-discharge relationships to forecast losses in fish biodiversity. Ecology.

[89] Abell R, Thieme ML, Revenga C, Bryer M, Kottelat M, Bogutskaya N, Coad B, Mandrak N, Balderas SC, Bussing W, Stiassny MLJ, Skelton P, Allen GR, Unmack P, Naseka A, Ng R, Sindorf N, Robertson J, Armijo E, Higgins JV, Heibel TJ, Wikramanayake E, Olson D, López HL, Reis RE, Lundberg JG, Sabaj Pérez MH, Petry P (2008). Freshwater ecoregions of the world: a new map of biogeographic units for freshwater biodiversity conservation. BioScience.

[90] Myhre, G. et al. In: Climate Change 2013: The Physical Science Basis. Contribution of Working Group I to the Fifth Assessment Report of the Intergovernmental Panel on Climate Change. (Cambridge University Press, 2013).

[91] Verones F (2020). LC-IMPACT: A regionalized life cycle damage assessment method. J. Ind. Ecol..

[92] Thematic Mapping API. *World Borders Dataset*. http://thematicmapping.org/downloads/world_borders.php (2009).

[93] ESRI. *ArcGis Desktop—ArcMap Version 10.8*. https://desktop.arcgis.com/en/arcmap/ (2020).

