# Supplementary material for: Controlling biodiversity impacts of future global hydropower reservoirs by strategic site selection
Source: Sci Rep. 2020 Dec 11;10:21777. doi: 10.1038/s41598-020-78444-6 (PMC7732980; doi:10.1038/s41598-020-78444-6)
Supplement: Supplementary file 1 — Supplementary Information. [file 41598_2020_78444_MOESM1_ESM.docx]

**Controlling** **biodiversity impacts of** **future global hydropower reservoirs by** **strategic site selection**

*Supplementary Information*

*Martin Dorber^*, †^, Anders Arvesen^†^, David Gernaat^a,b^, Francesca Verones^†^*

^†^Department of Energy and Process Engineering, NTNU, Høgskoleringen 5, 7491 Trondheim, Norway

^a^ PBL - Netherlands Environment Assessment Agency, The Hague, The Netherlands

^b^ Copernicus Institute of Sustainable Development, Utrecht University, Utrecht, The Netherlands

20 pages

23 Figures

0 Tables

Table of Contents

[S1: Hydropower locations 3](#_Toc50726719)

[S2: Land occupation 4](#_Toc50726720)

[S3: Water consumption 5](#_Toc50726721)

[S4: Methane emissions 6](#_Toc50726722)

[S5: Land occupation biodiversity impact 7](#_Toc50726723)

[S6: Water consumption biodiversity impact 8](#_Toc50726724)

[S7 Terrestrial biodiversity-impact-wise best hydropower reservoirs 9](#_Toc50726725)

[S8 Aquatic biodiversity-impact-wise best hydropower reservoirs 14](#_Toc50726726)

[S9: Overall biodiversity impact 19](#_Toc50726727)

[S10: References used within the supplementary materials 20](#_Toc50726728)

# S1: Hydropower locations

In this study, we performed a spatially explicit assessment of terrestrial and aquatic biodiversity impacts of possible 1,956 possible future hydropower reservoirs. The possible new hydropower reservoirs sites were identified by Gernaat et al.^1^ and are shown in Figure S1.


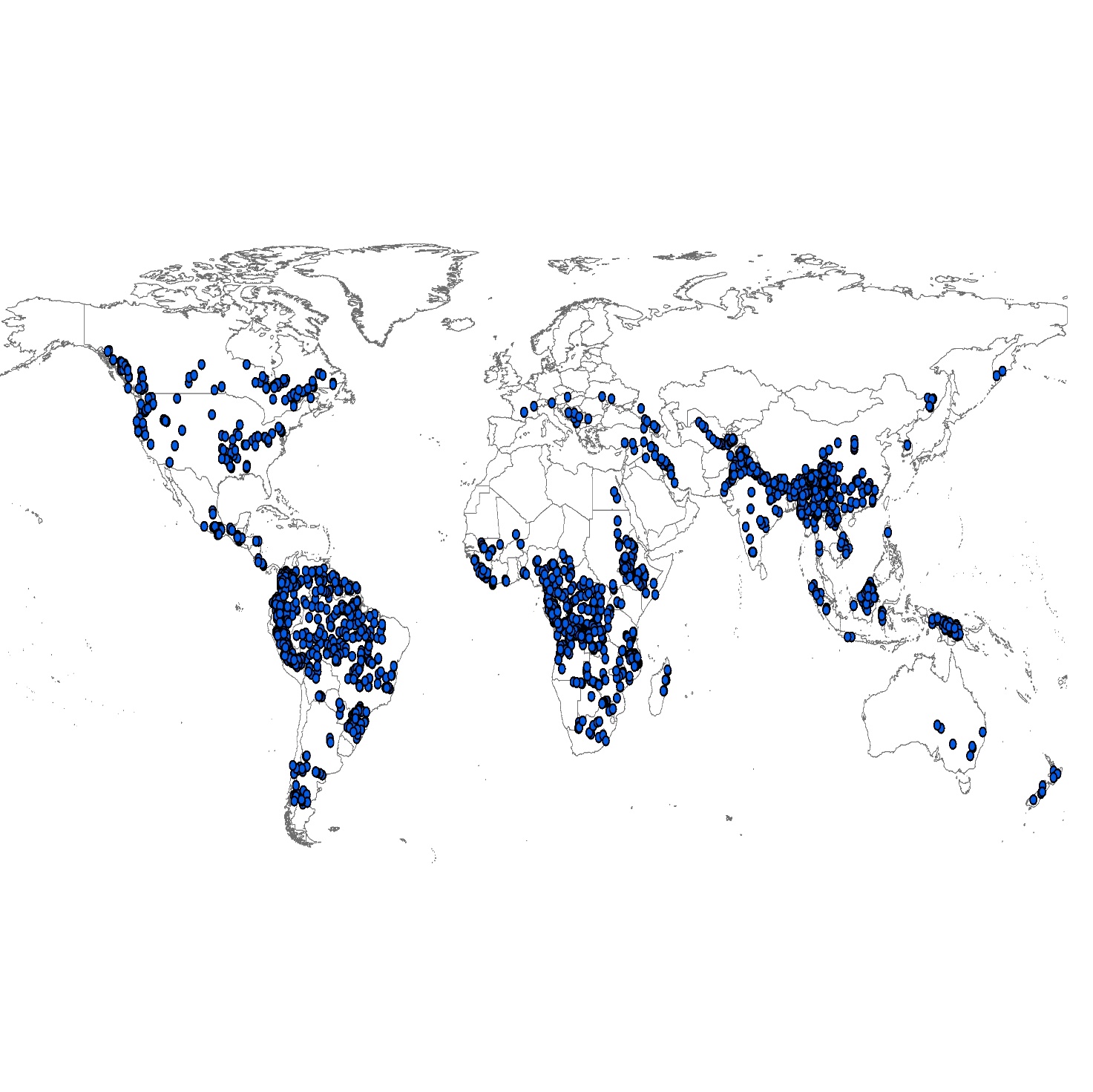


Figure S1: The blue points show the location of the 1,956 possible new hydropower reservoirs, identified by Gernaat et al.^1^, for which we assessed a terrestrial or aquatic biodiversity impact. World borders obtained from ref^3^ and created with ArcMap 10.8 (ref^4^).

# S2: Land occupation

The land occupation of all 1955 potential future hydropower reservoirs varies between 0.00007

m^2^*y/kWh and 9.49 m^2^*y/kWh, with an average of 0.068 m^2^*y/kWh (Figure S2). The land occupation depends on the inundated land area and the amount of electricity produced. The inundated land area itself influenced by the reservoir surface area and the present natural water area.


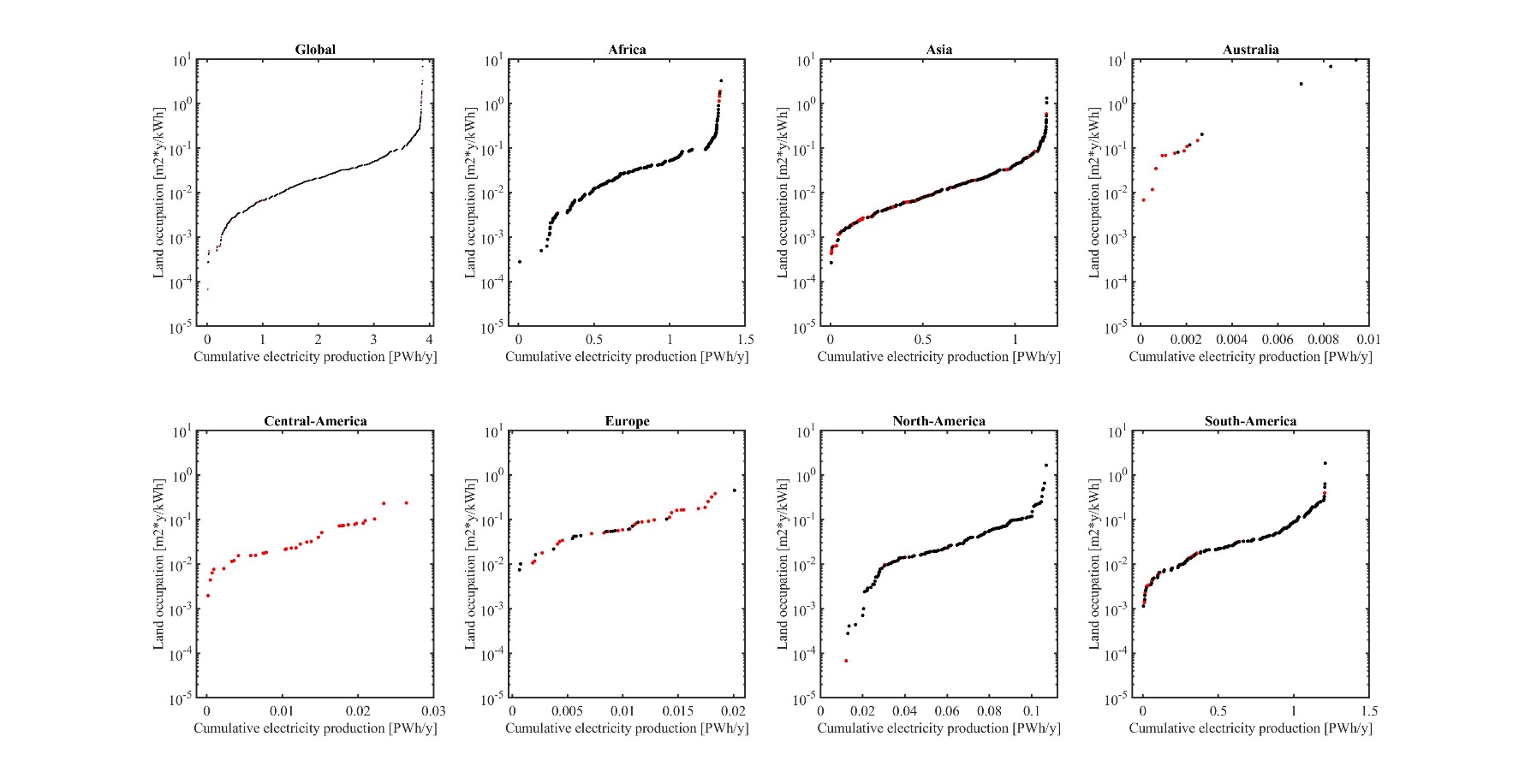


Figure S2: Land occupation in m^2^*y/kWh for each hydropower reservoir, compared to the compared cumulative electricity production in PWh yr^-1^. The scale of the y-axis is logarithmic. Red points = reservoirs located in a biodiversity hotspot^2^; Black points: not located in a biodiversity hotspot^2^.

# S3: Water consumption

We were able to calculate a water consumption value for 1941 hydropower reservoirs. For the remaining 14 reservoirs, no water consumption value could be calculated, due to missing evaporation values. The water consumption varies between 0.0002 m^3^/kWh and 25.2 m^3^/kWh, with an average of 0.10 m^3^/kWh (Figure S3). The water consumption depends on the inundated land area, the present land cover type and the climatic conditions.


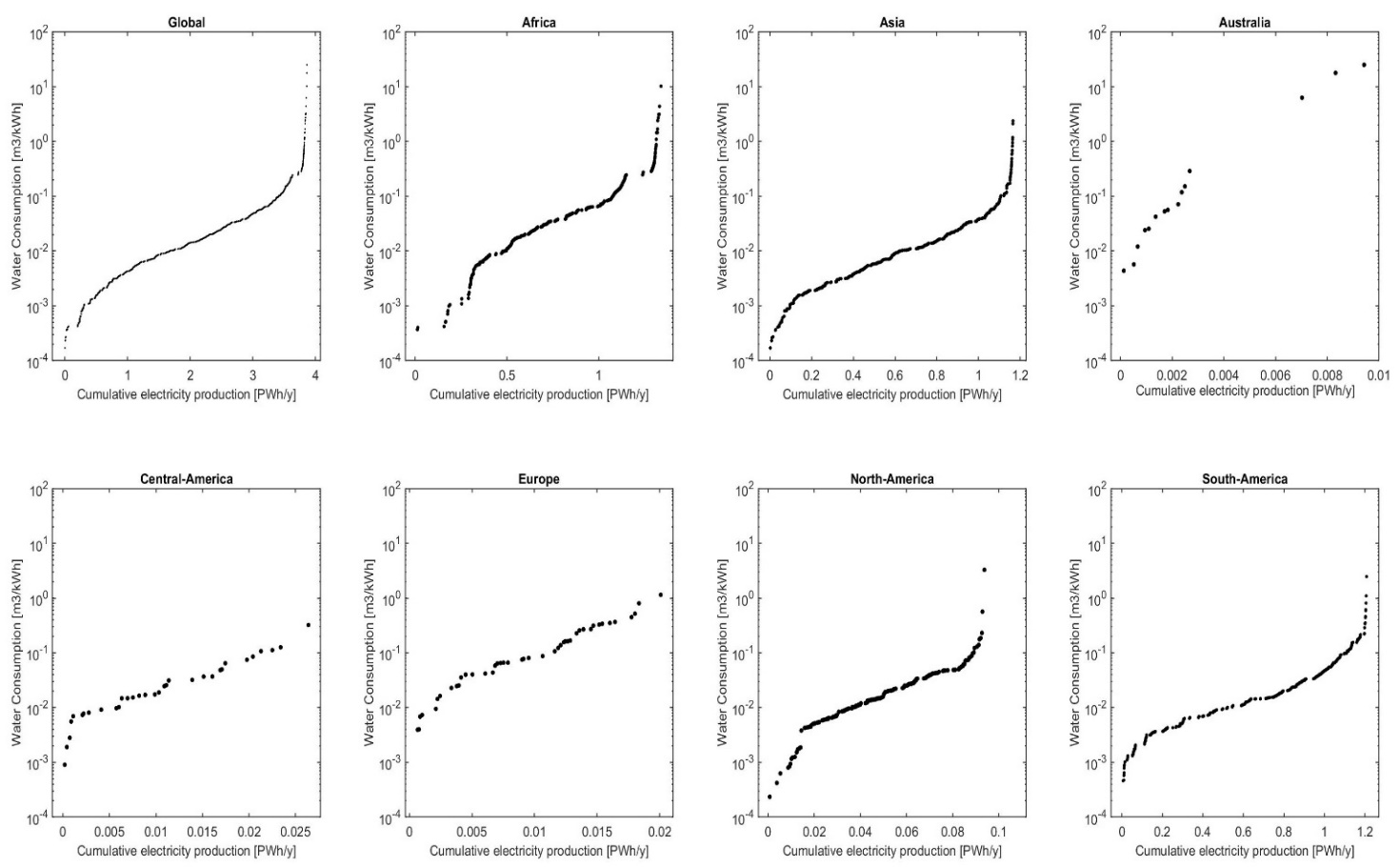


Figure S3: Water consumption in m^3^/kWh for each hydropower reservoir, compared to the compared cumulative electricity production in PWh yr^-1^. The scale of the y-axis is logarithmic.

# S4: Methane emissions

We were able to calculate methane emissions for 1940 hydropower reservoirs. For the remaining 15 reservoirs no methane emissions could be calculated, due to missing temperature values. The methane emissions vary between 0.0005 g/kWh and 1211 g/kWh, with an average of 3.38 g/kWh (Figure S4). The methane emission depends on the area to electricity ration and the maximum air temperature.

**
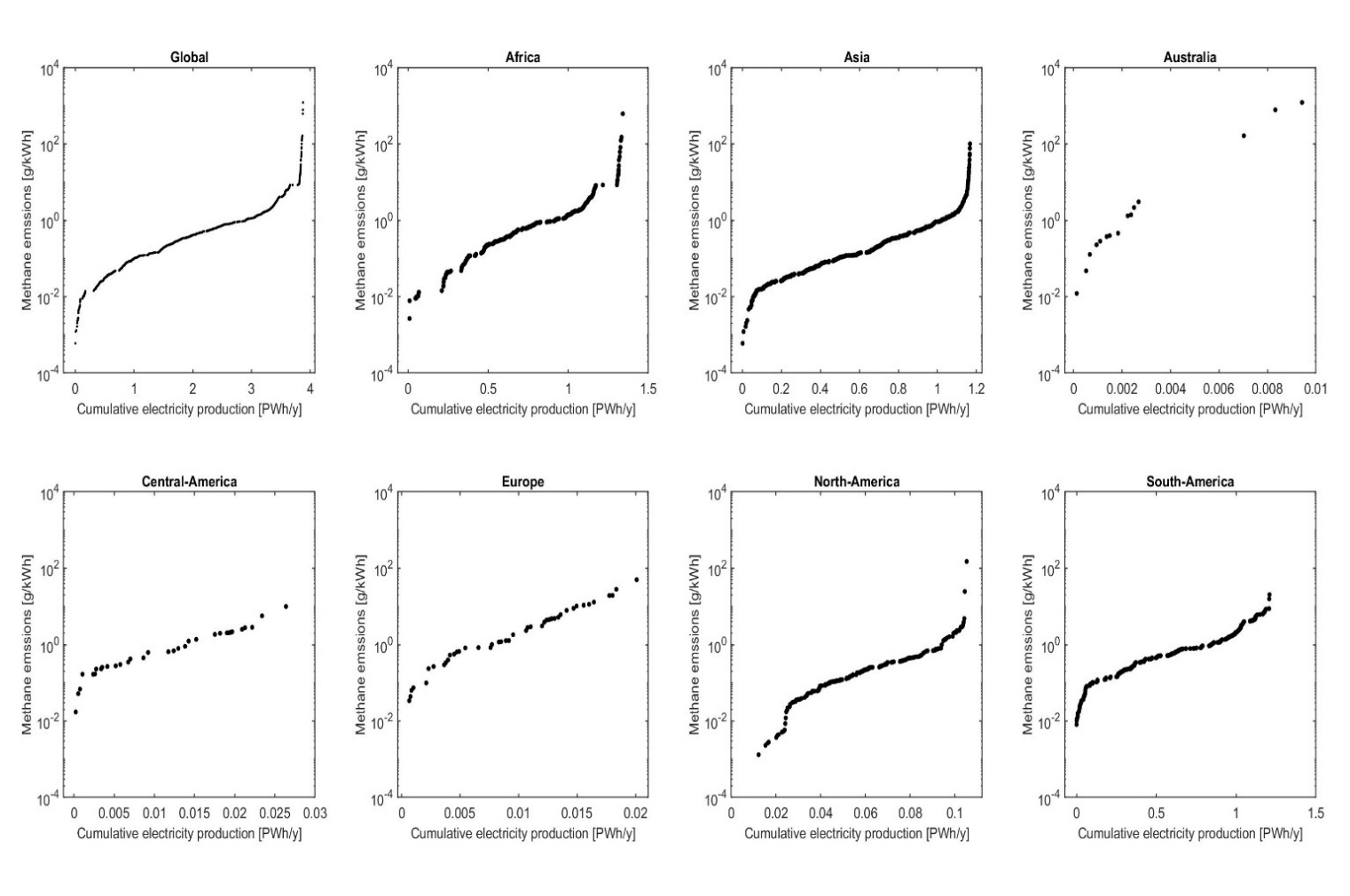
**

Figure S4: Methane emissions in g/kWh for each hydropower reservoir, compared to the compared cumulative electricity production in PWh yr^-1^. The scale of the y-axis is logarithmic.

# S5: Land occupation biodiversity impact

The terrestrial biodiversity impact of land occupation varies between 0 PDF*y/kWh and 4.44E-12 PDF*y/kWh (Figure S5). The biodiversity impact depends on the m^2^*y/kWh land occupation, inundated land use type, terrestrial ecoregion, and the global extinction probability (GEP) of the taxonomic groups present in the terrestrial ecoregion.

**
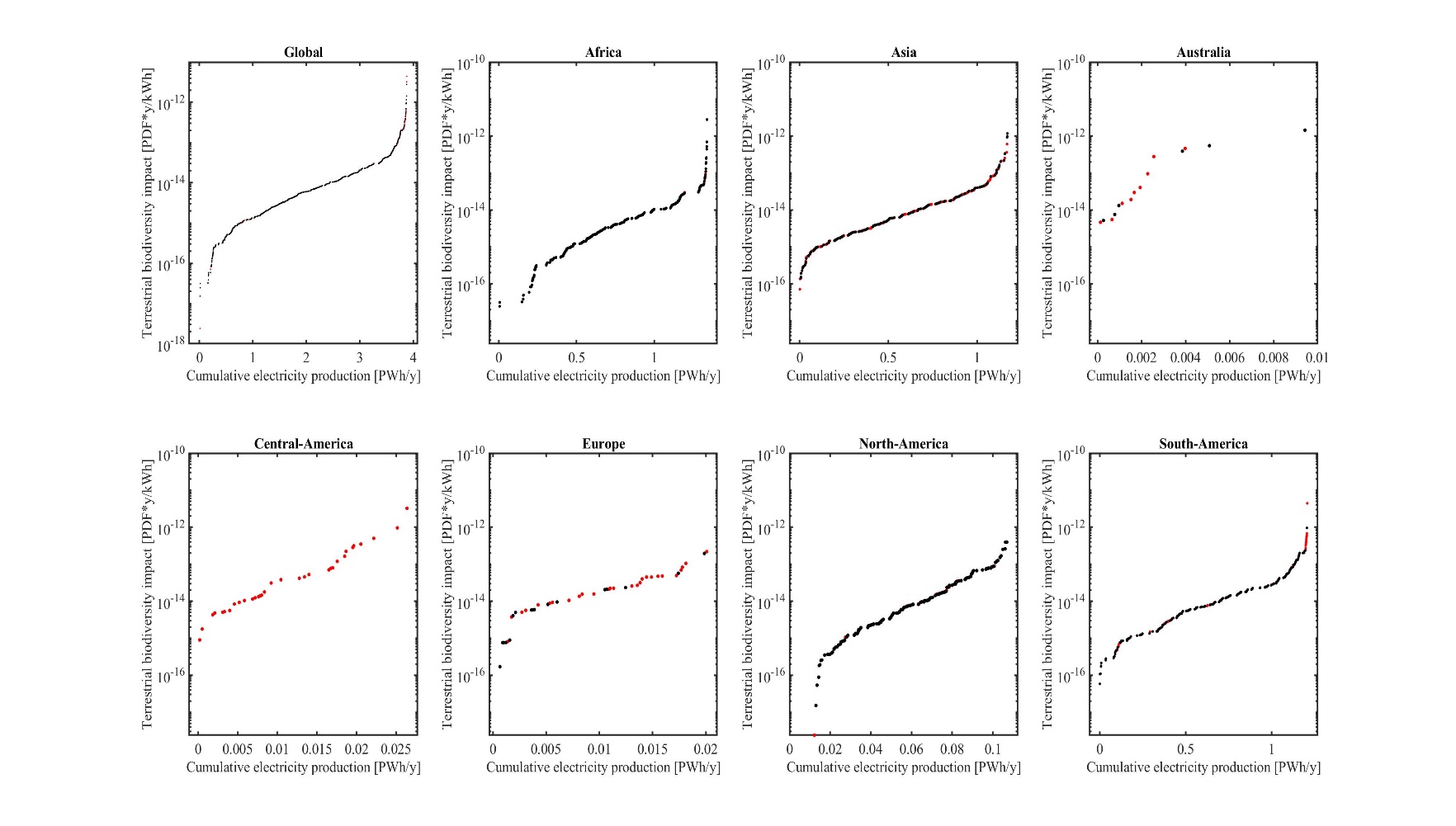
**

Figure S5: Biodiversity supply curve, showing the terrestrial biodiversity impact of land occupation in PDF*y/kWh, compared to the cumulative electricity production in PWh yr^-1^. The scale of the y-axis is logarithmic. Red points = reservoirs located in a biodiversity hotspot^2^; Black points: not located in a biodiversity hotspot^2^.

# S6: Water consumption biodiversity impact

The aquatic biodiversity impact of water consumption impact varies between 4.68E-13 PDF*y/kWh and 1.41E-19 PDF*y/kWh (Figure S6). The biodiversity impact depends on the land occupation, differences between PET and AET, climatic conditions, river discharge and GEP of the freshwater groups present in the freshwater ecoregion.

**
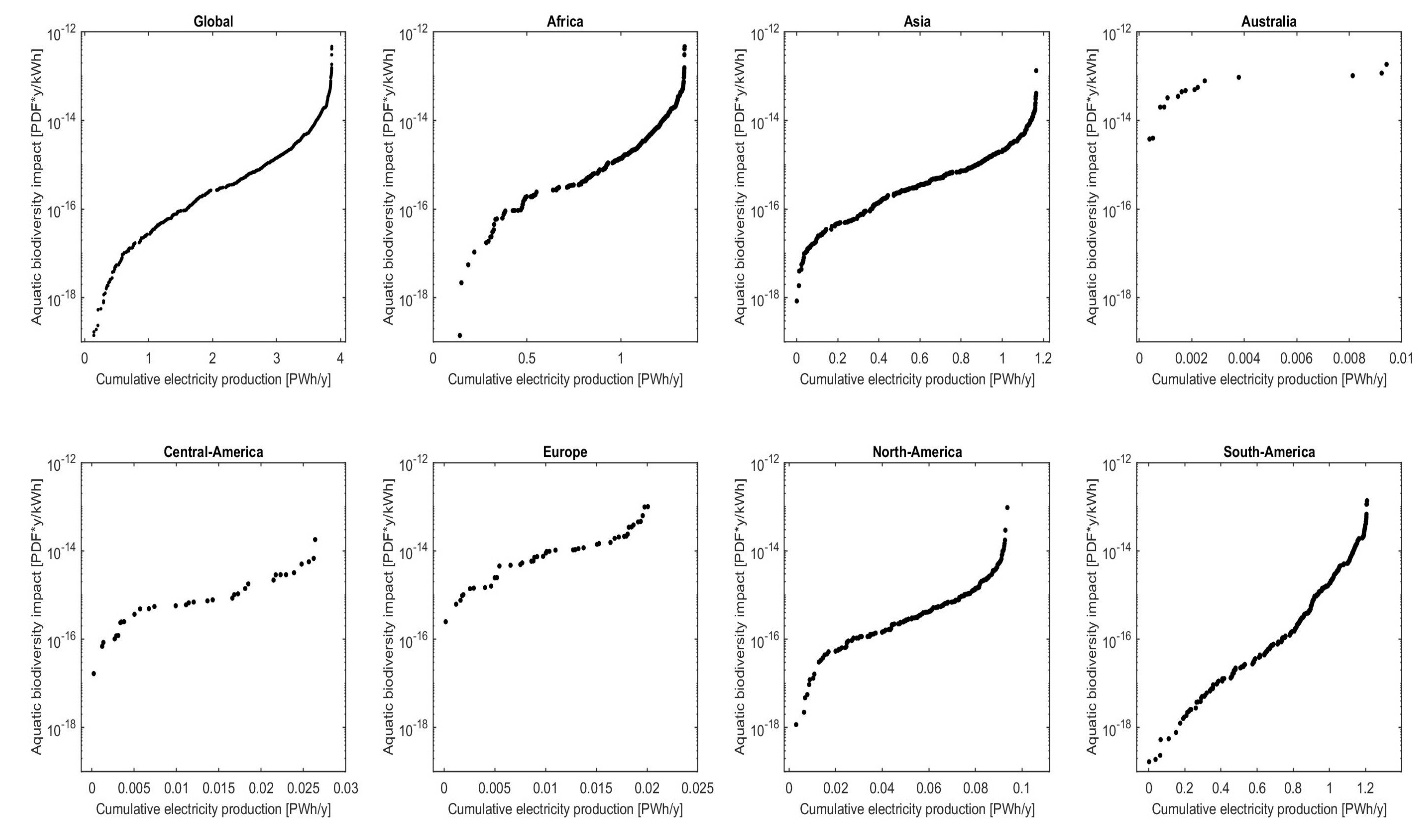
**

Figure S6: Biodiversity supply curve, showing the aquatic biodiversity impact of water consumption in PDF*y/kWh, compared to the cumulative electricity production in PWh yr^-1^. The scale of the y-axis is logarithmic.

# S7 Terrestrial biodiversity-impact-wise best hydropower reservoirs


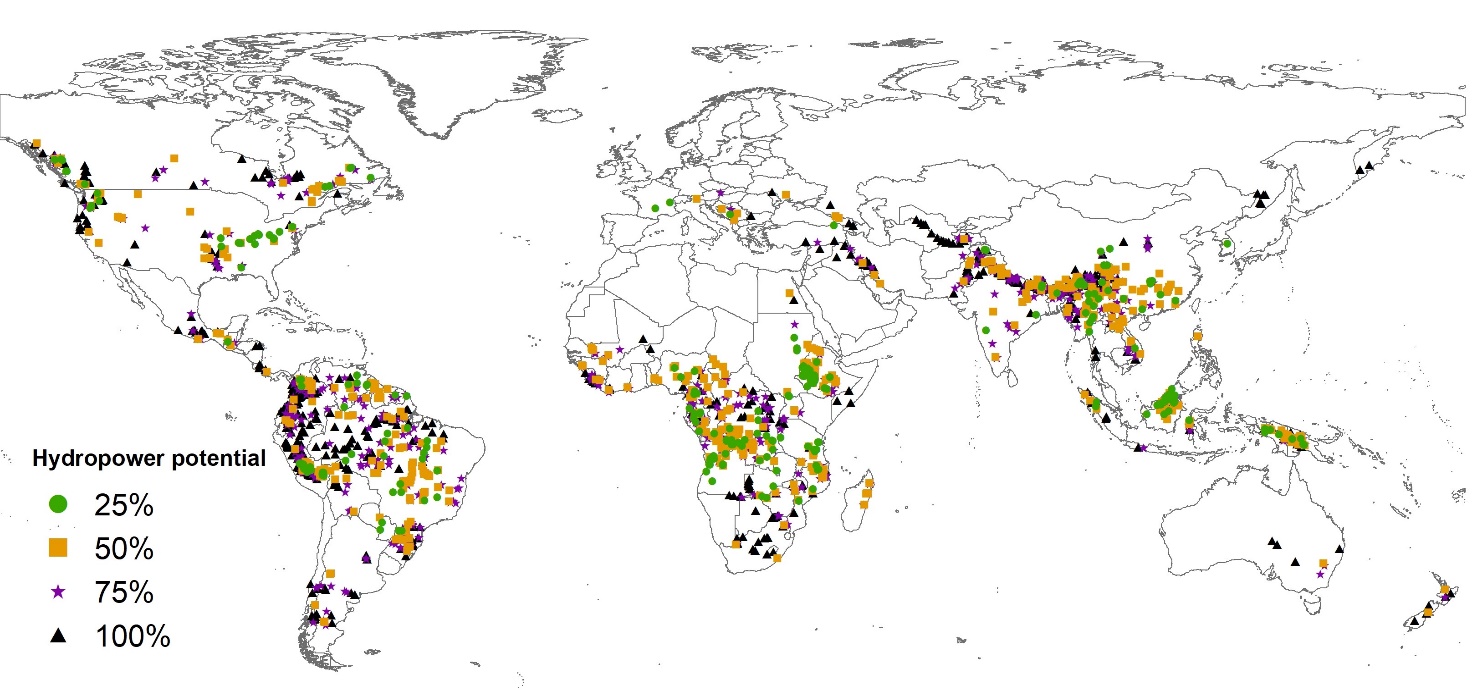


Figure S7: Location of the terrestrial biodiversity-impact-wise best hydropower reservoirs, that could be built to reach 25%, 50%, 75% and 100% of the global hydropower potential. (i.e. the best are the green dots, followed by the yellow squares and purple stars. The black triangles should be avoided if possible). World borders obtained from ref^3^ and created with ArcMap 10.8 (ref^4^).


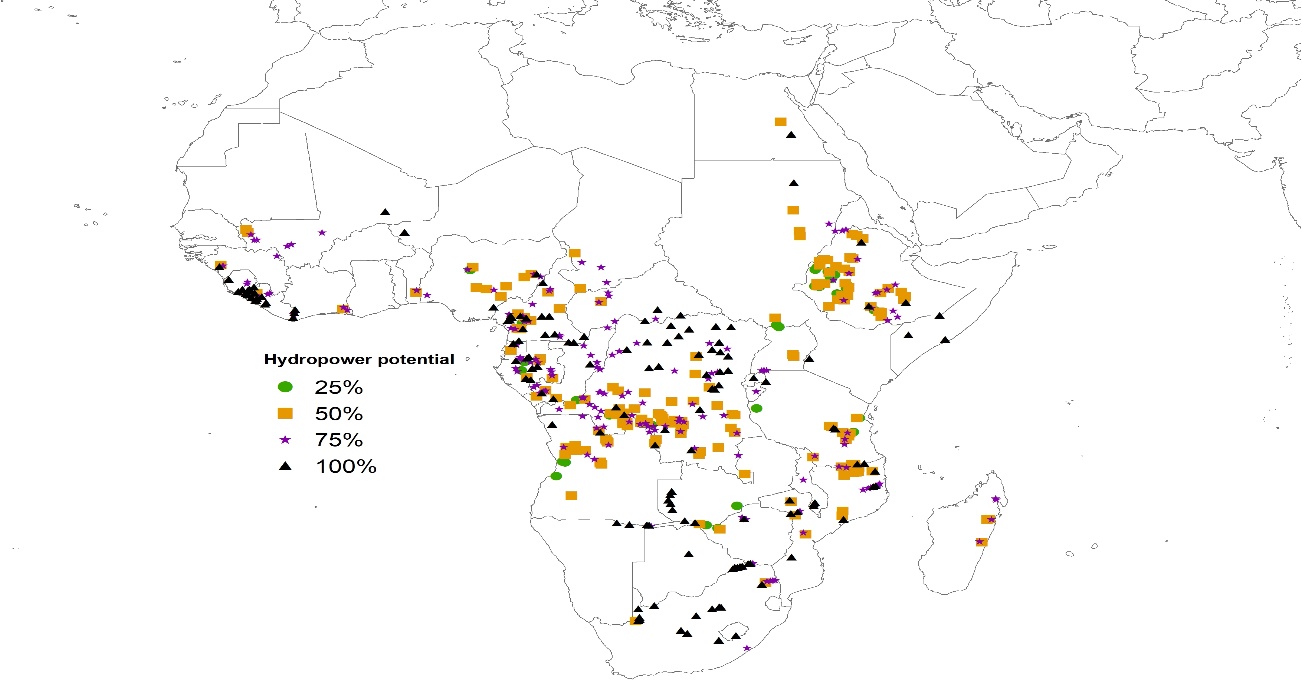


Figure S8: Location of the terrestrial biodiversity-impact-wise best hydropower reservoirs, that could be built to reach 25%, 50%, 75% and 100% of the African hydropower potential. World borders obtained from ref^3^ and created with ArcMap 10.8 (ref^4^).


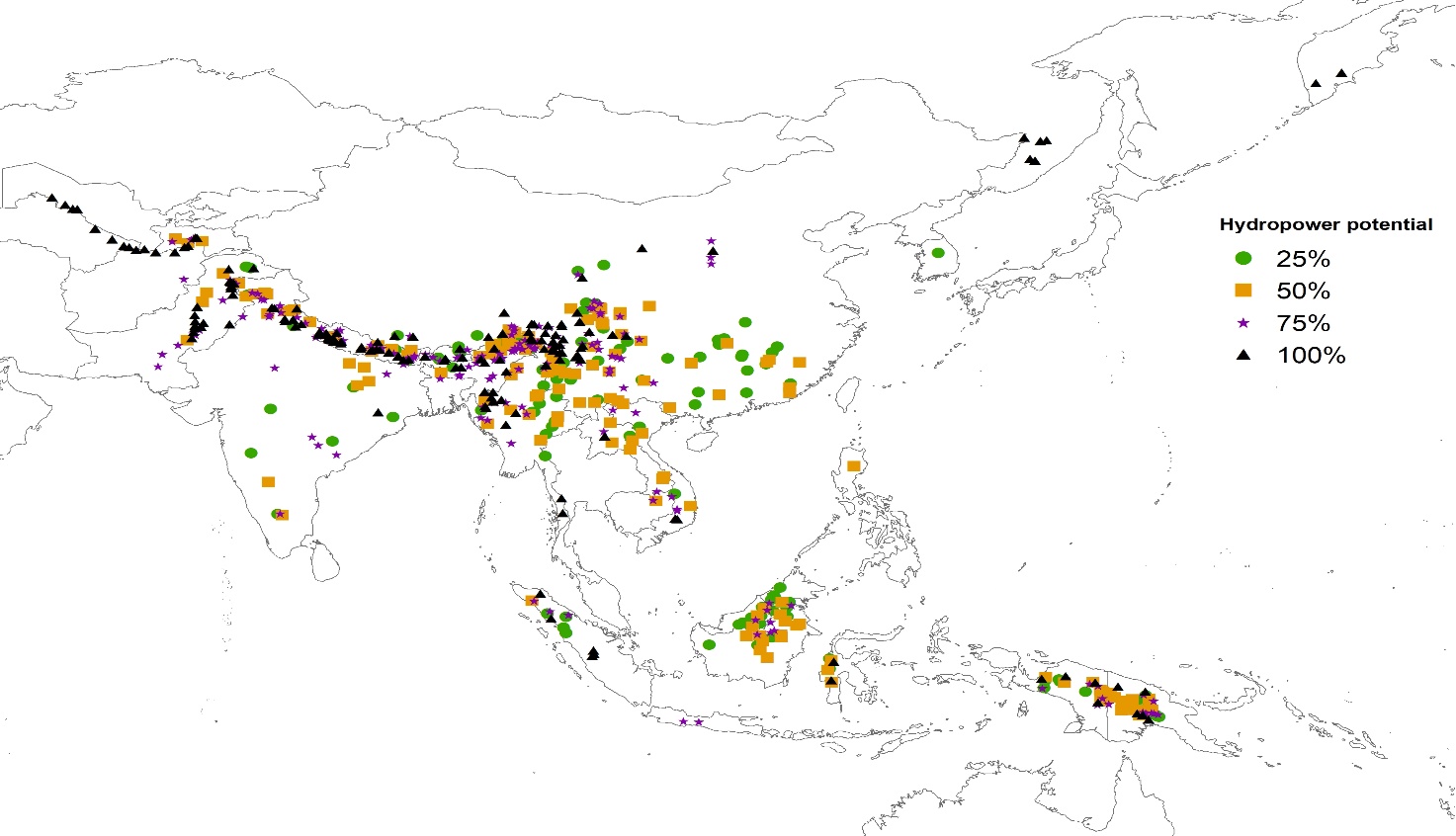


Figure S9: Location of the terrestrial biodiversity-impact-wise best hydropower reservoirs, that could be built to reach 25%, 50%, 75% and 100% of the Asian hydropower potential. World borders obtained from ref^3^ and created with ArcMap 10.8 (ref^4^).


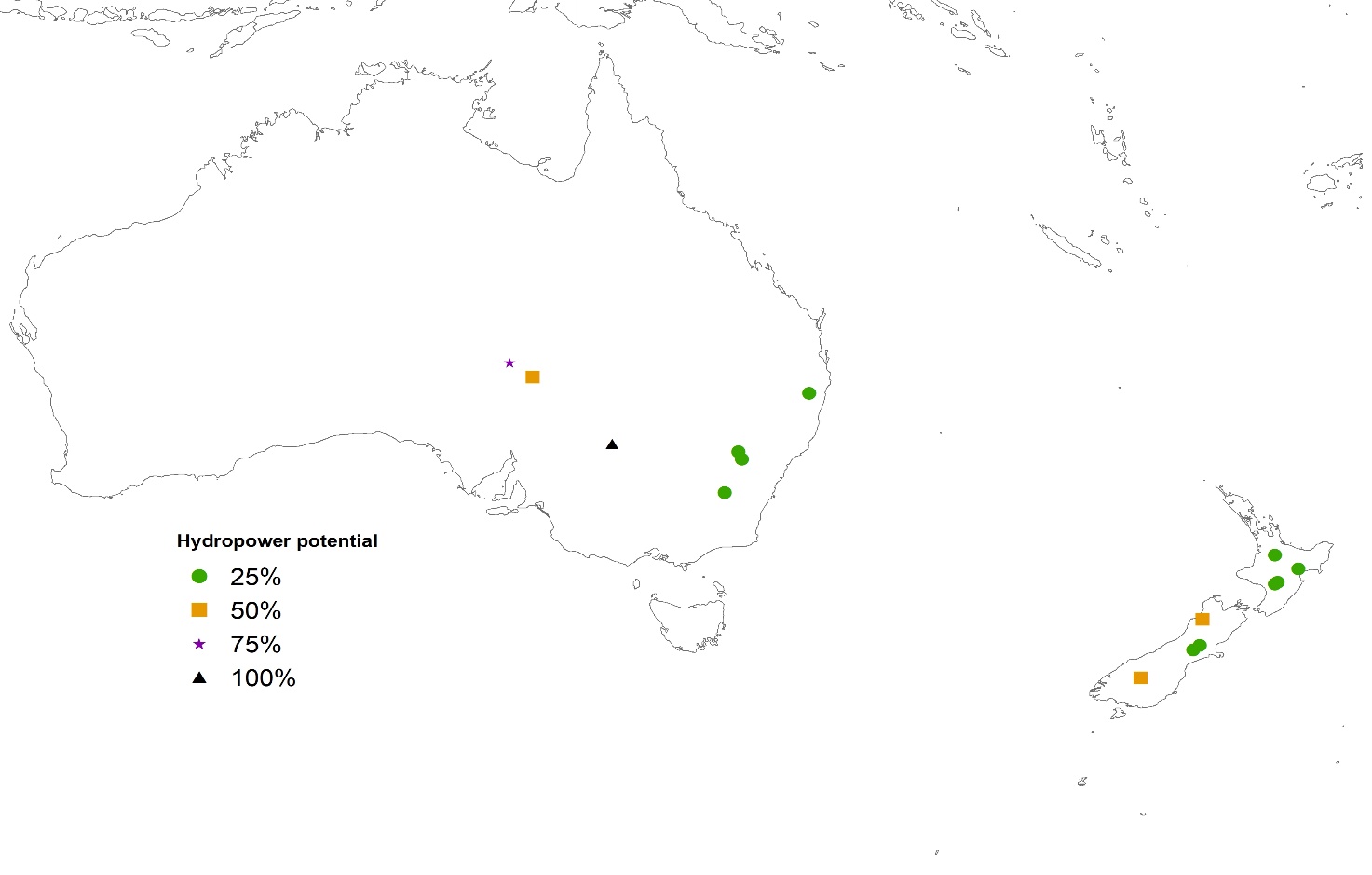


Figure S10: Location of the terrestrial biodiversity-impact-wise best hydropower reservoirs, that could be built to reach 25%, 50%, 75% and 100% of the Australian hydropower potential. World borders obtained from ref^3^ and created with ArcMap 10.8 (ref^4^).


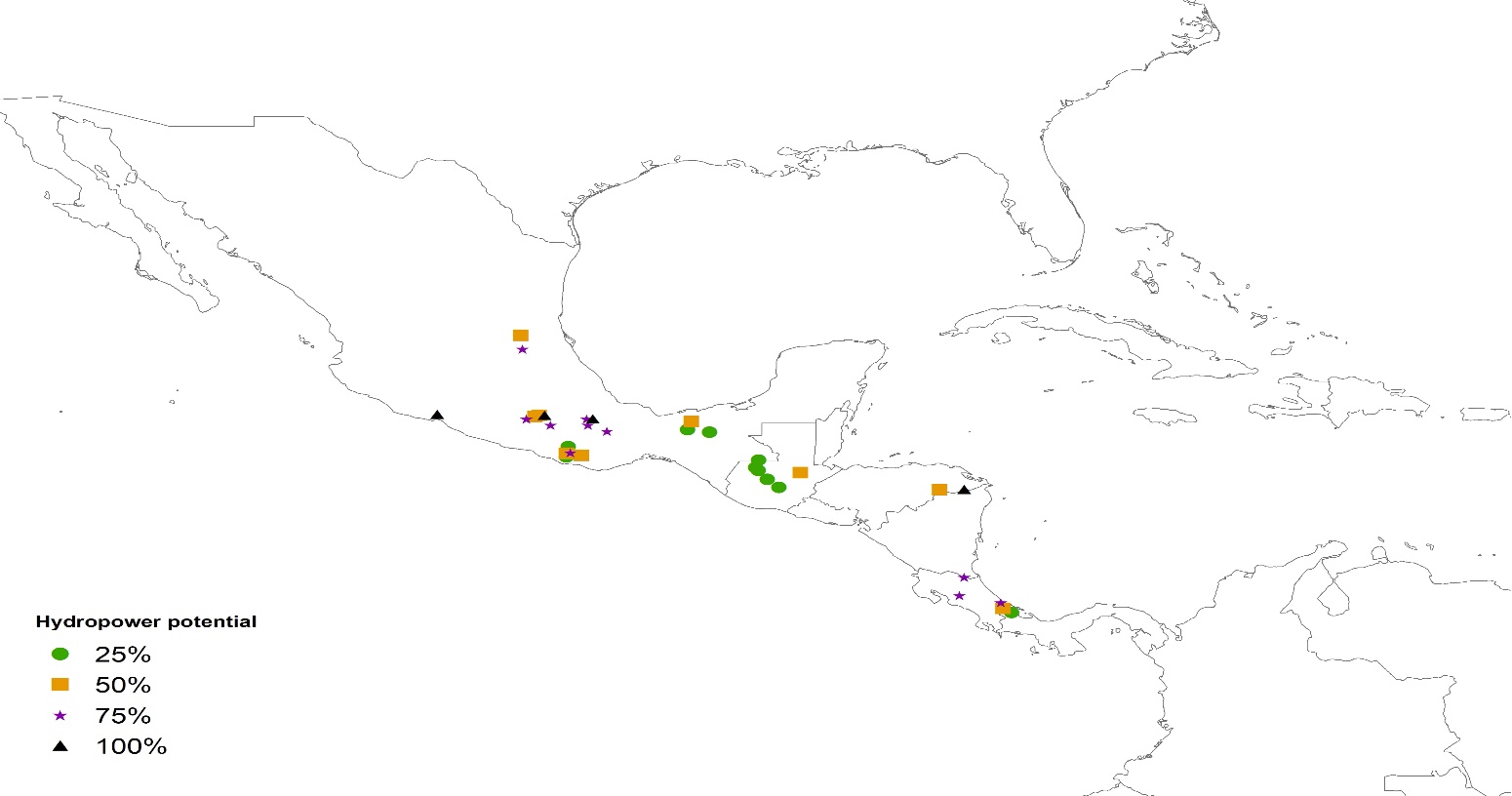


Figure S11: Location of the terrestrial biodiversity-impact-wise best hydropower reservoirs, that could be built to reach 25%, 50%, 75% and 100% of the Central-American hydropower potential. World borders obtained from ref^3^ and created with ArcMap 10.8 (ref^4^).


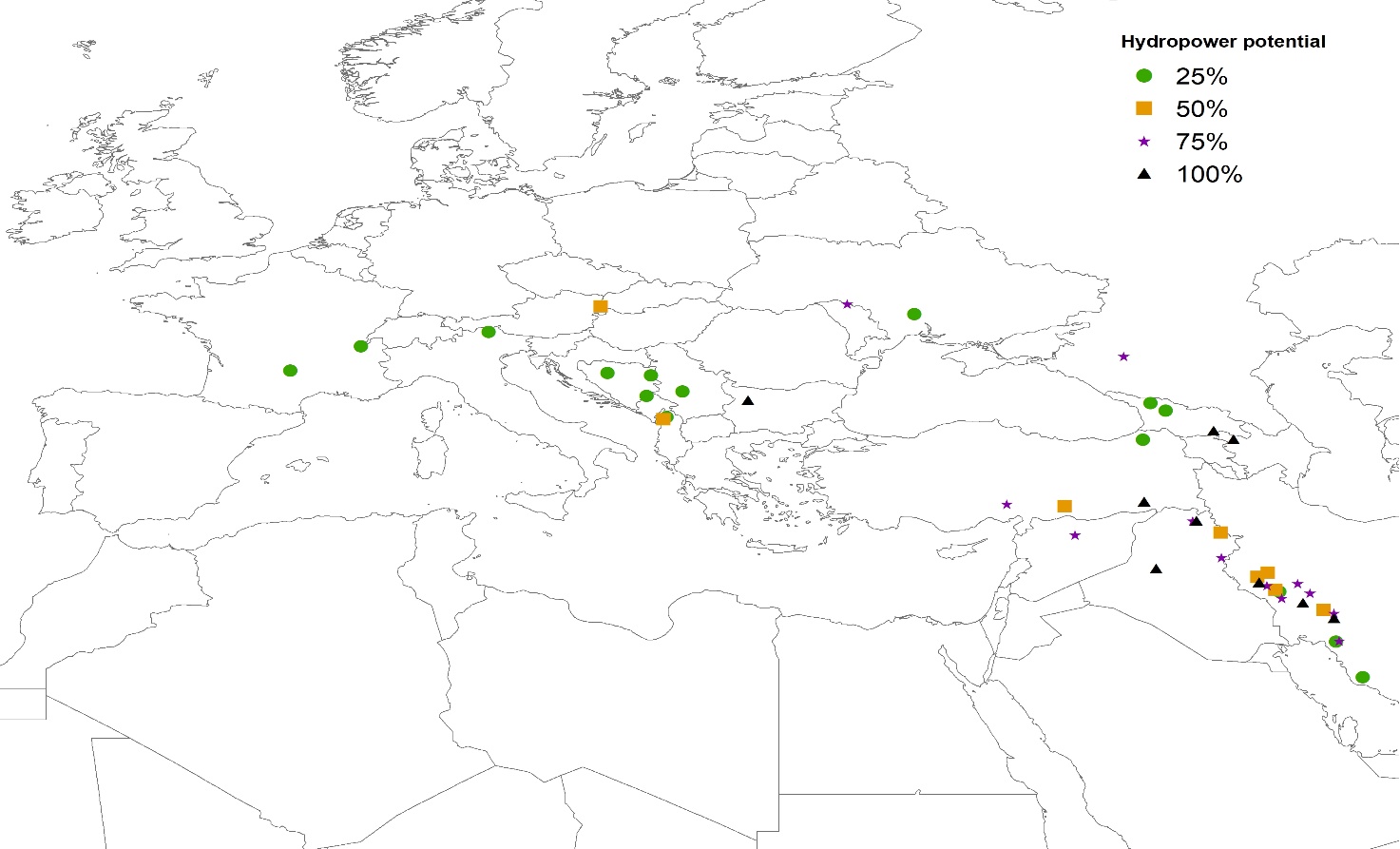


Figure S12: Location of the terrestrial biodiversity-impact-wise best hydropower reservoirs, that could be built to reach 25%, 50%, 75% and 100% of the European hydropower potential. World borders obtained from ref^3^ and created with ArcMap 10.8 (ref^4^).


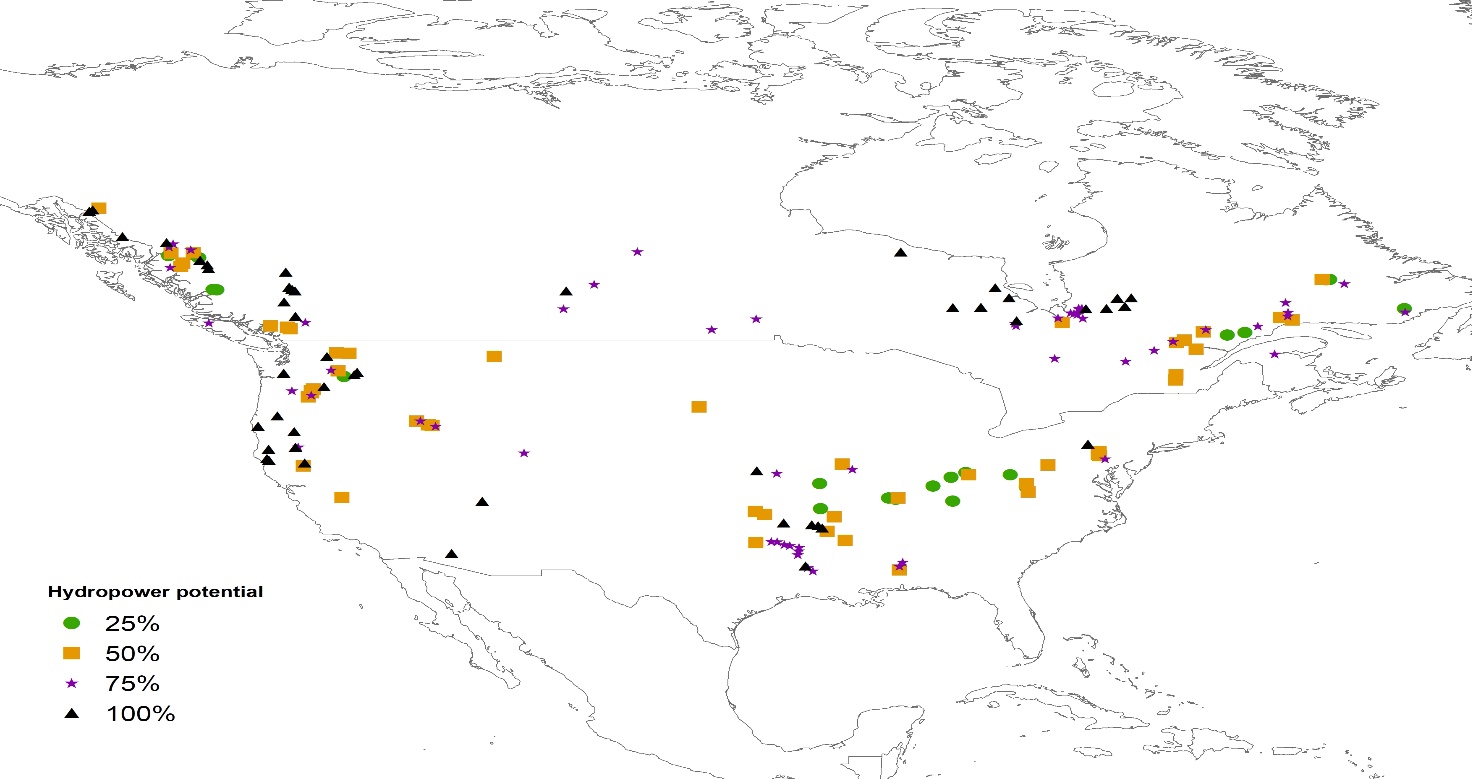


Figure S13: Location of the terrestrial biodiversity-impact-wise best hydropower reservoirs, that could be built to reach 25%, 50%, 75% and 100% of the North American hydropower potential. World borders obtained from ref^3^ and created with ArcMap 10.8 (ref^4^).


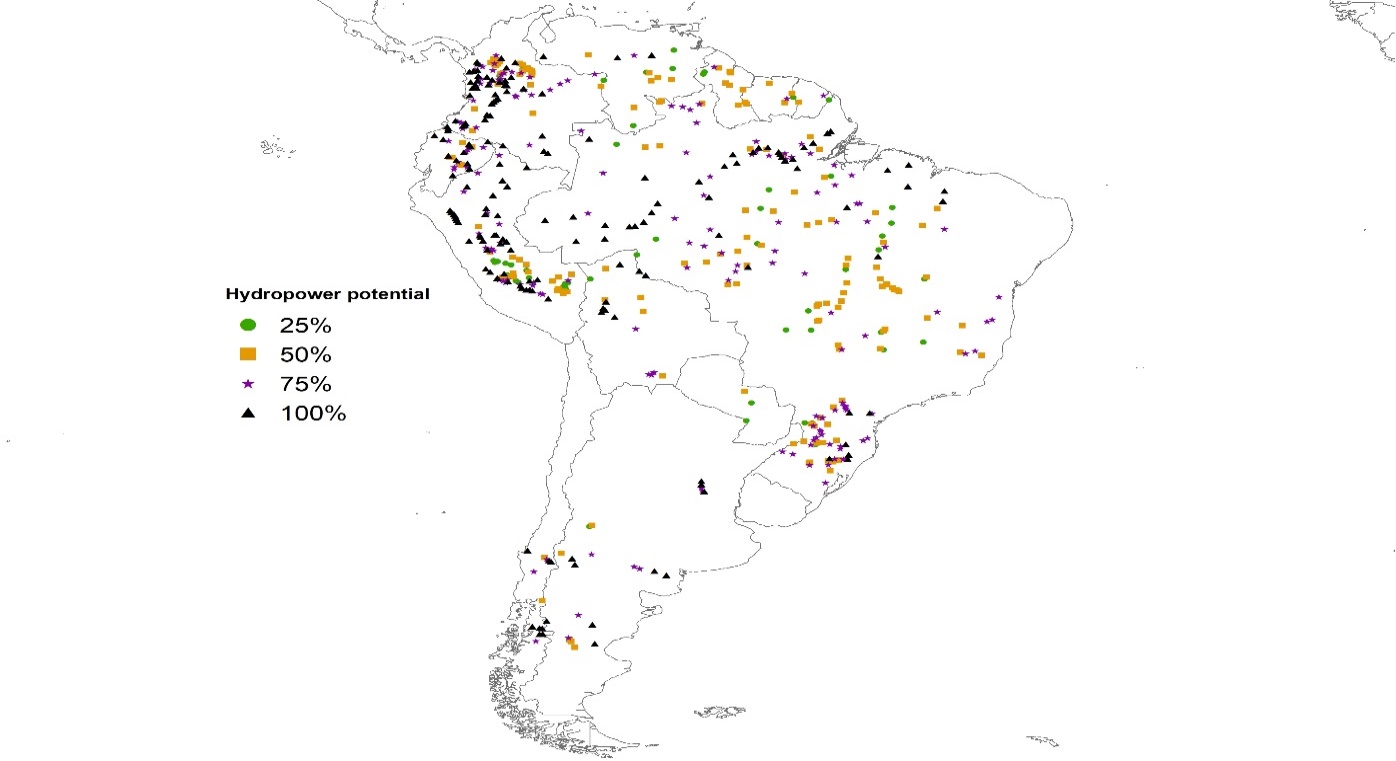


Figure S14: Location of the terrestrial biodiversity-impact-wise best hydropower reservoirs, that could be built to reach 25%, 50%, 75% and 100% of the South American hydropower potential. World borders obtained from ref^3^ and created with ArcMap 10.8 (ref^4^).

# S8 Aquatic biodiversity-impact-wise best hydropower reservoirs


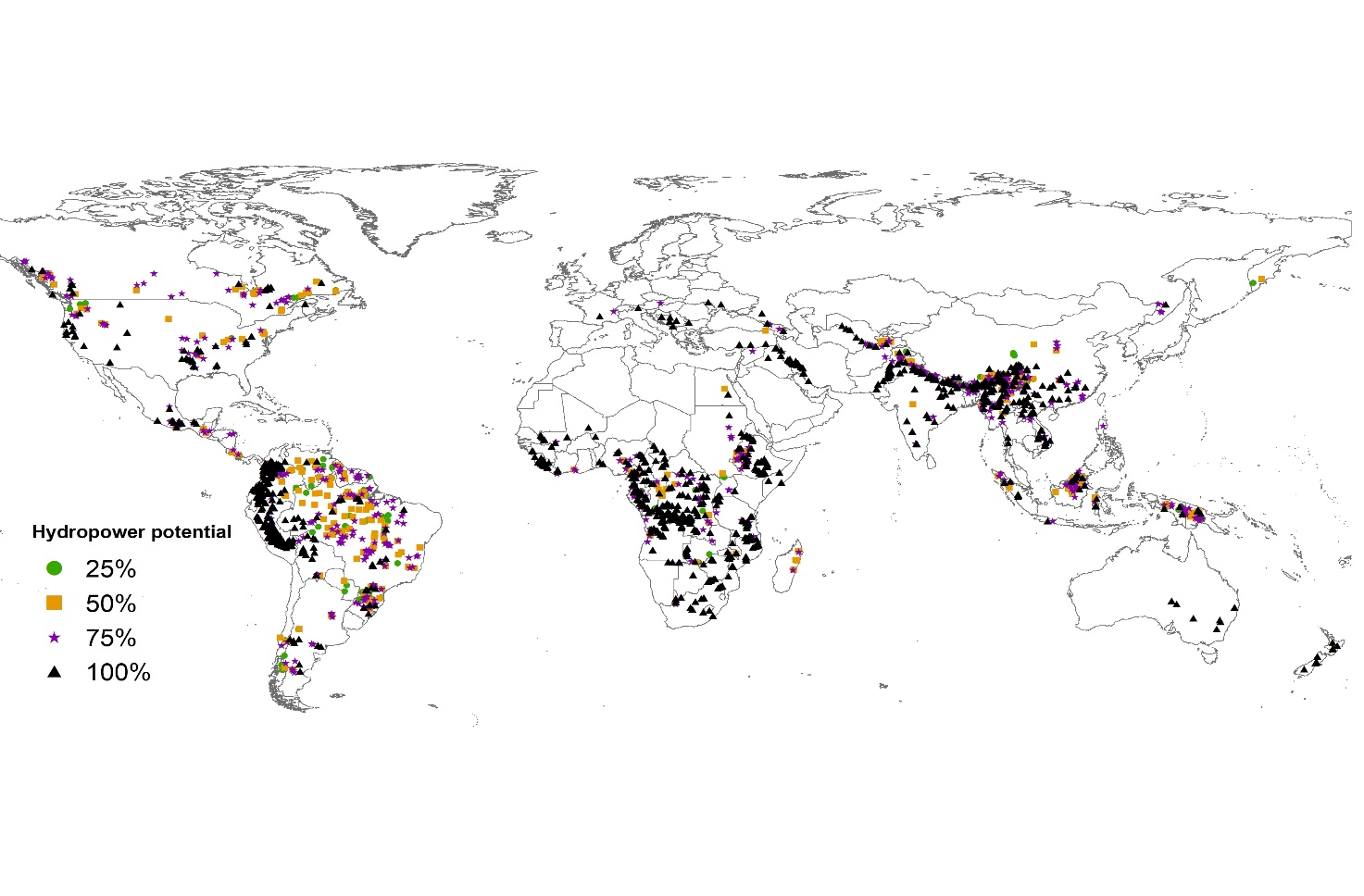


Figure S15: Location of the aquatic biodiversity-impact-wise best hydropower reservoirs, that could be built to reach 25%, 50%, 75% and 100% of the global hydropower potential. (i.e. the best are the green dots, followed by the yellow squares and purple stars. The black triangles should be avoided if possible). World borders obtained from ref^3^ and created with ArcMap 10.8 (ref^4^).


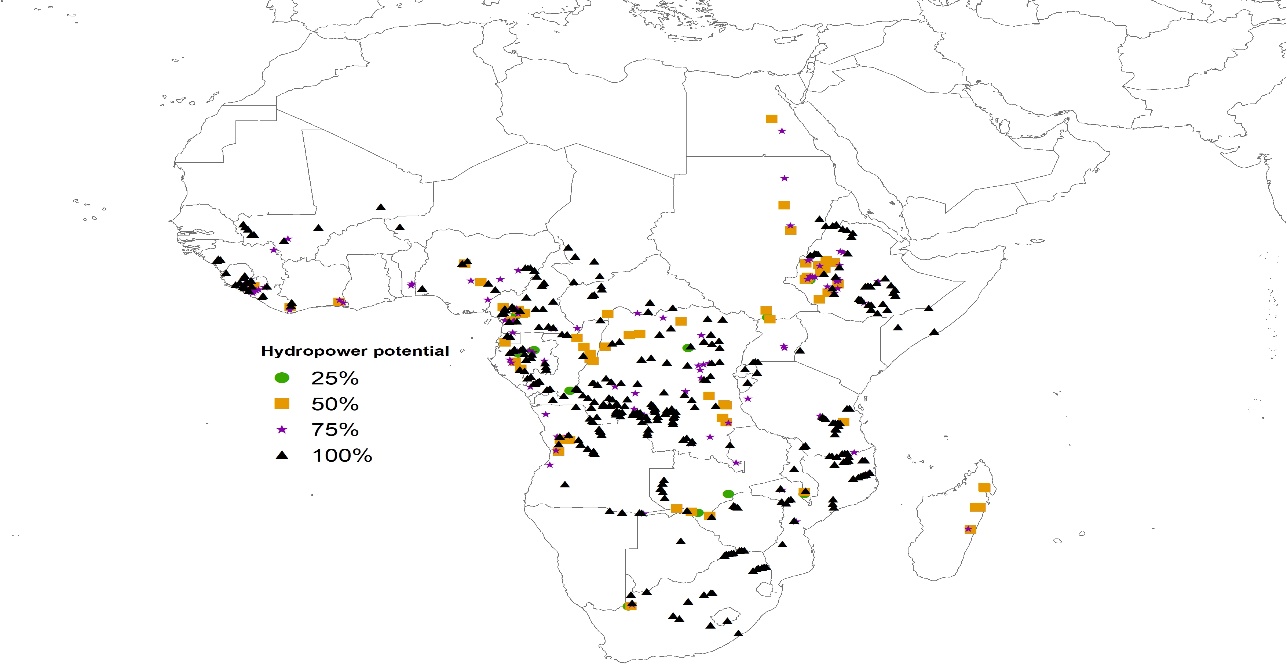


Figure S16: Location of the terrestrial biodiversity-impact-wise best hydropower reservoirs, that could be built to reach 25%, 50%, 75% and 100% of the African hydropower potential. World borders obtained from ref^3^ and created with ArcMap 10.8 (ref^4^).


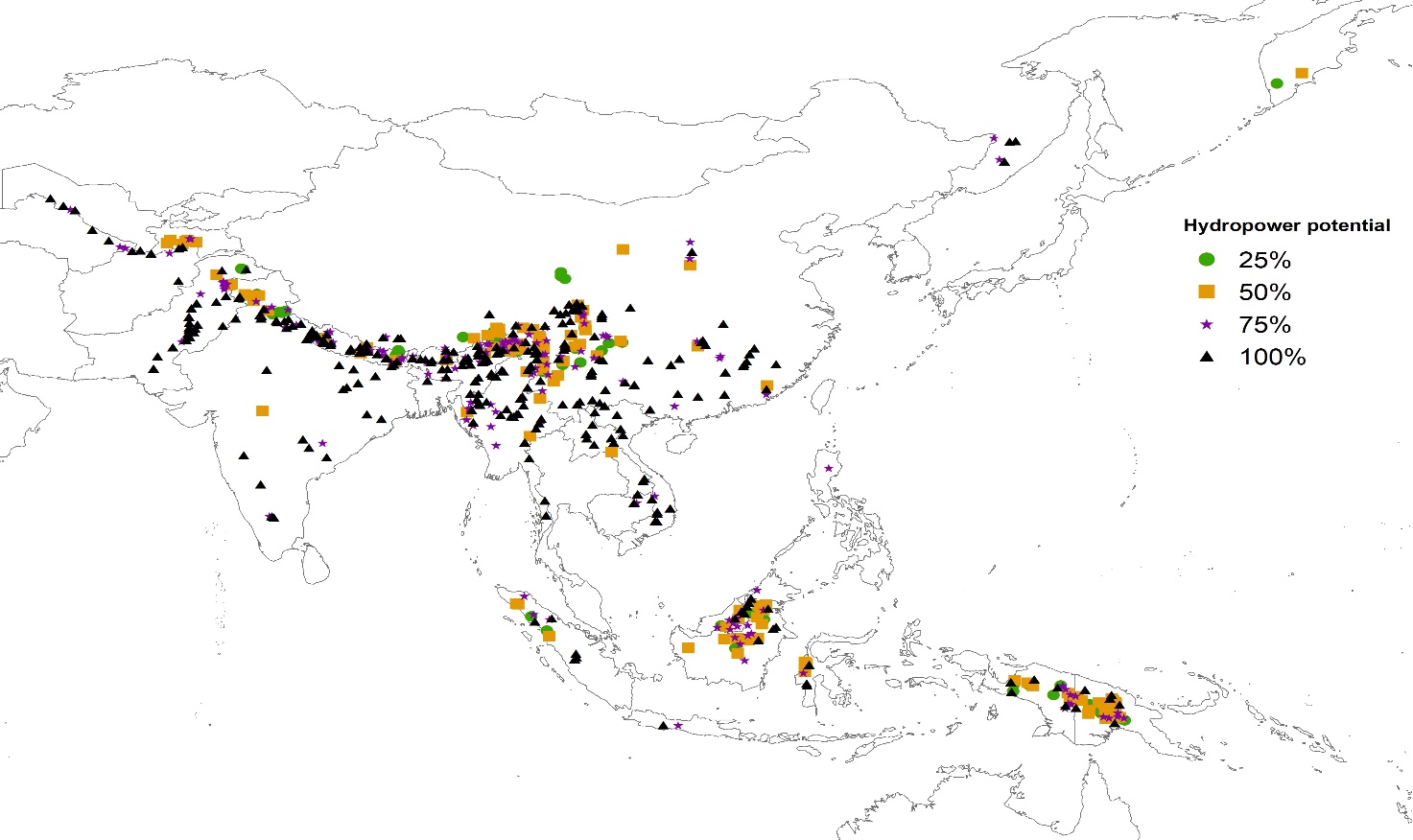


Figure S17: Location of the aquatic biodiversity-impact-wise best hydropower reservoirs, that could be built to reach 25%, 50%, 75% and 100% of the Asian hydropower potential. World borders obtained from ref^3^ and created with ArcMap 10.8 (ref^4^).


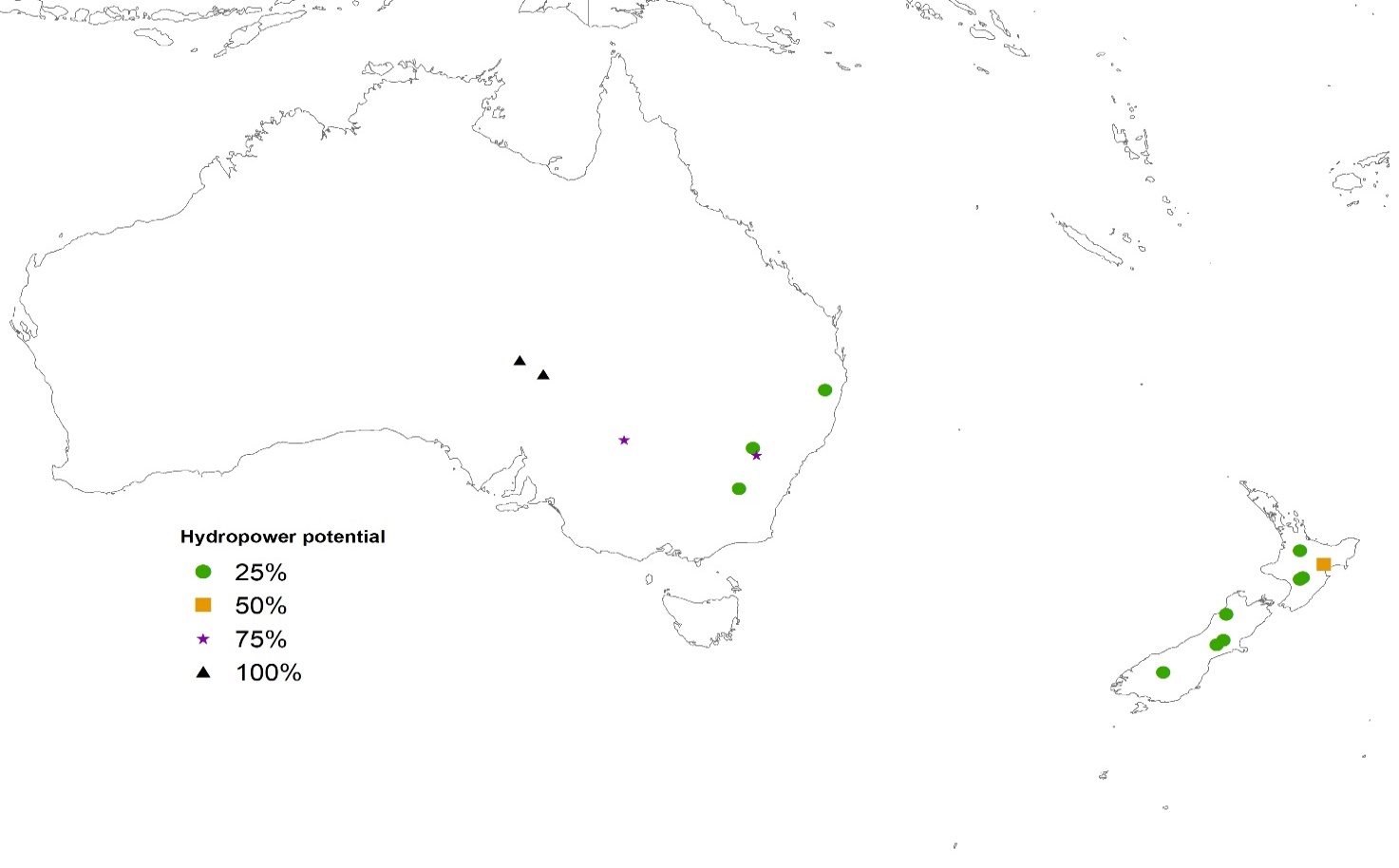


Figure S18: Location of the aquatic biodiversity-impact-wise best hydropower reservoirs, that could be built to reach 25%, 50%, 75% and 100% of the Australian hydropower potential. World borders obtained from ref^3^ and created with ArcMap 10.8 (ref^4^).


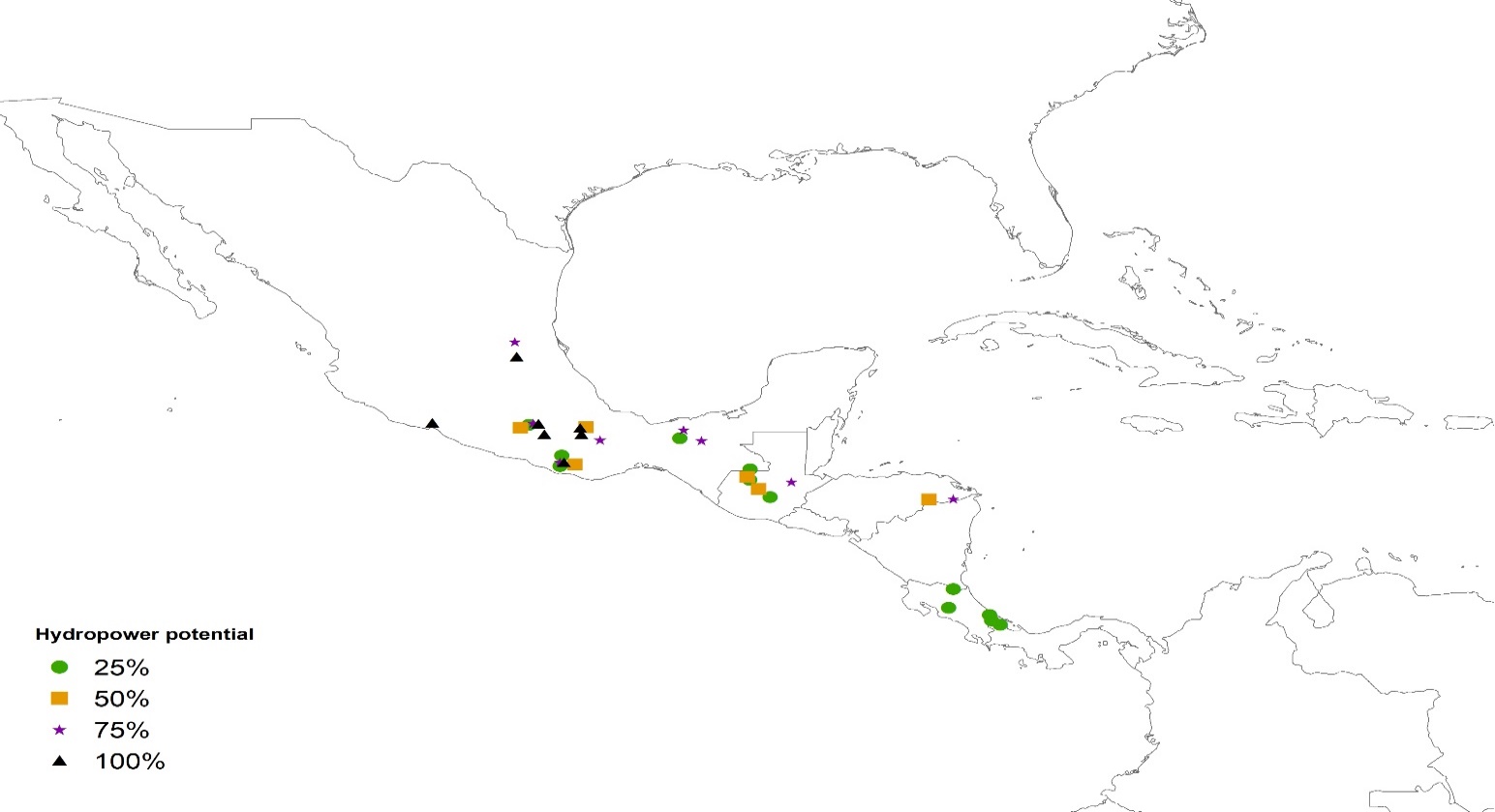


Figure S19: Location of the aquatic biodiversity-impact-wise best hydropower reservoirs, that could be built to reach 25%, 50%, 75% and 100% of the Central American hydropower potential. World borders obtained from ref^3^ and created with ArcMap 10.8 (ref^4^).


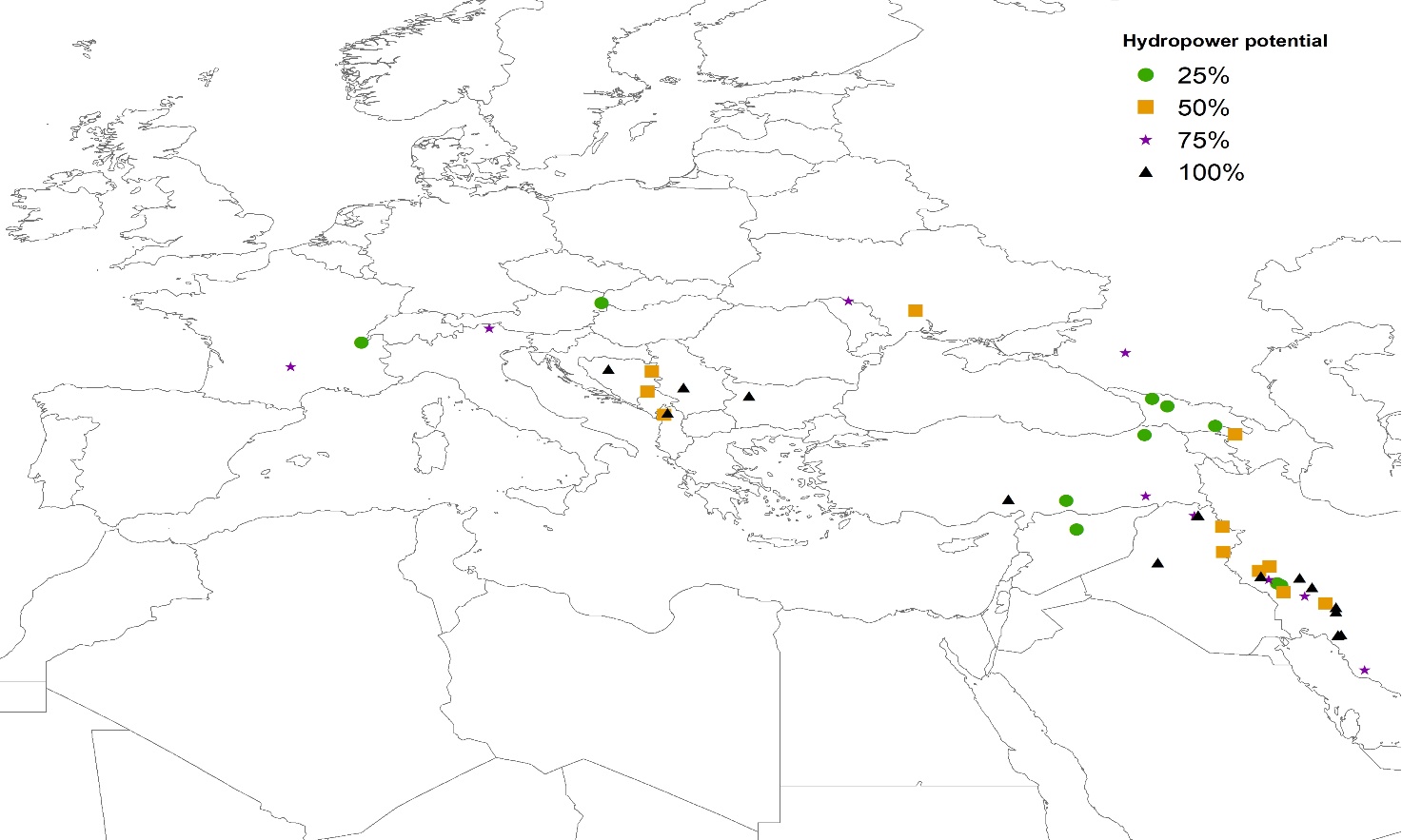


Figure S20: Location of the aquatic biodiversity-impact-wise best hydropower reservoirs, that could be built to reach 25%, 50%, 75% and 100% of the European hydropower potential. World borders obtained from ref^3^ and created with ArcMap 10.8 (ref^4^).


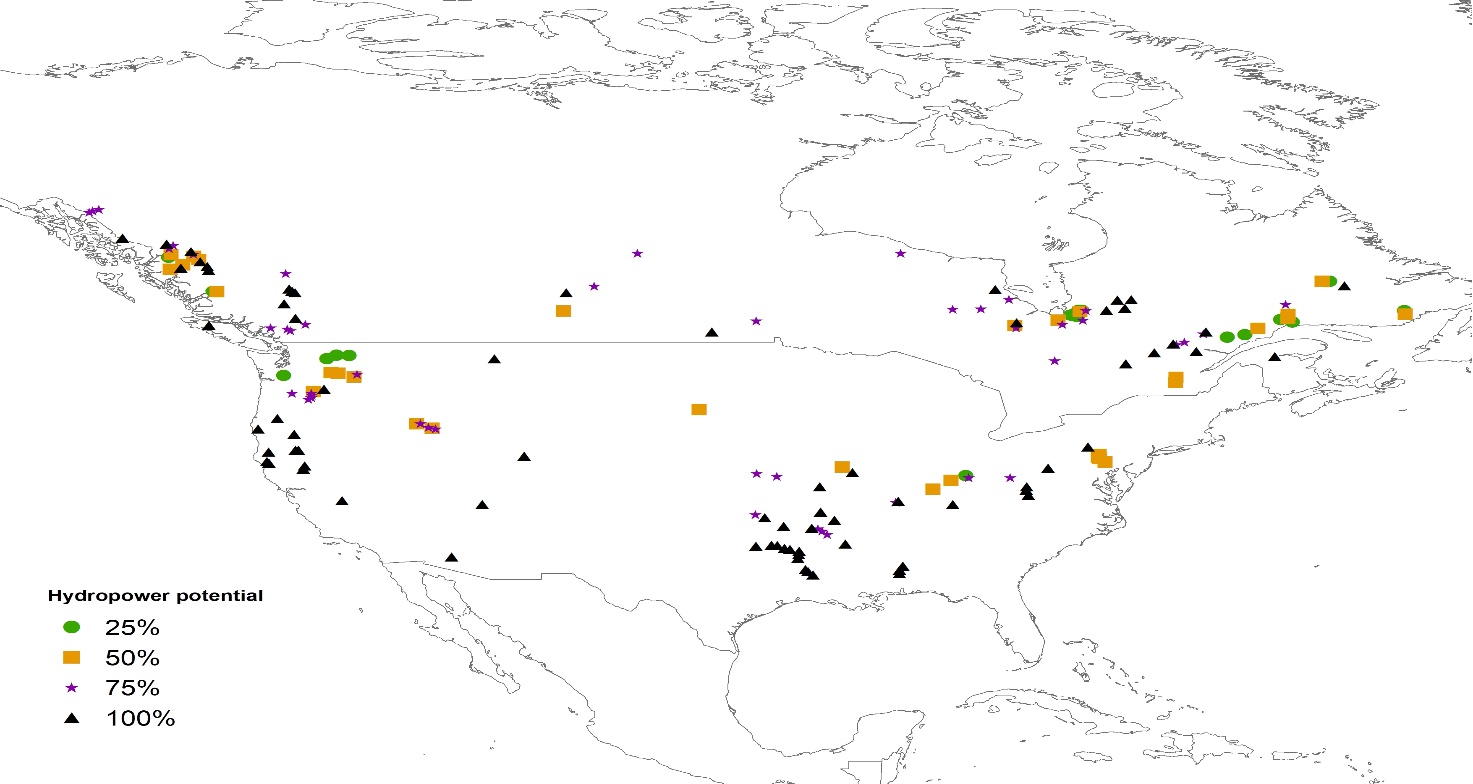


Figure S21: Location of the aquatic biodiversity-impact-wise best hydropower reservoirs, that could be built to reach 25%, 50%, 75% and 100% of the North American hydropower potential. World borders obtained from ref^3^ and created with ArcMap 10.8 (ref^4^).


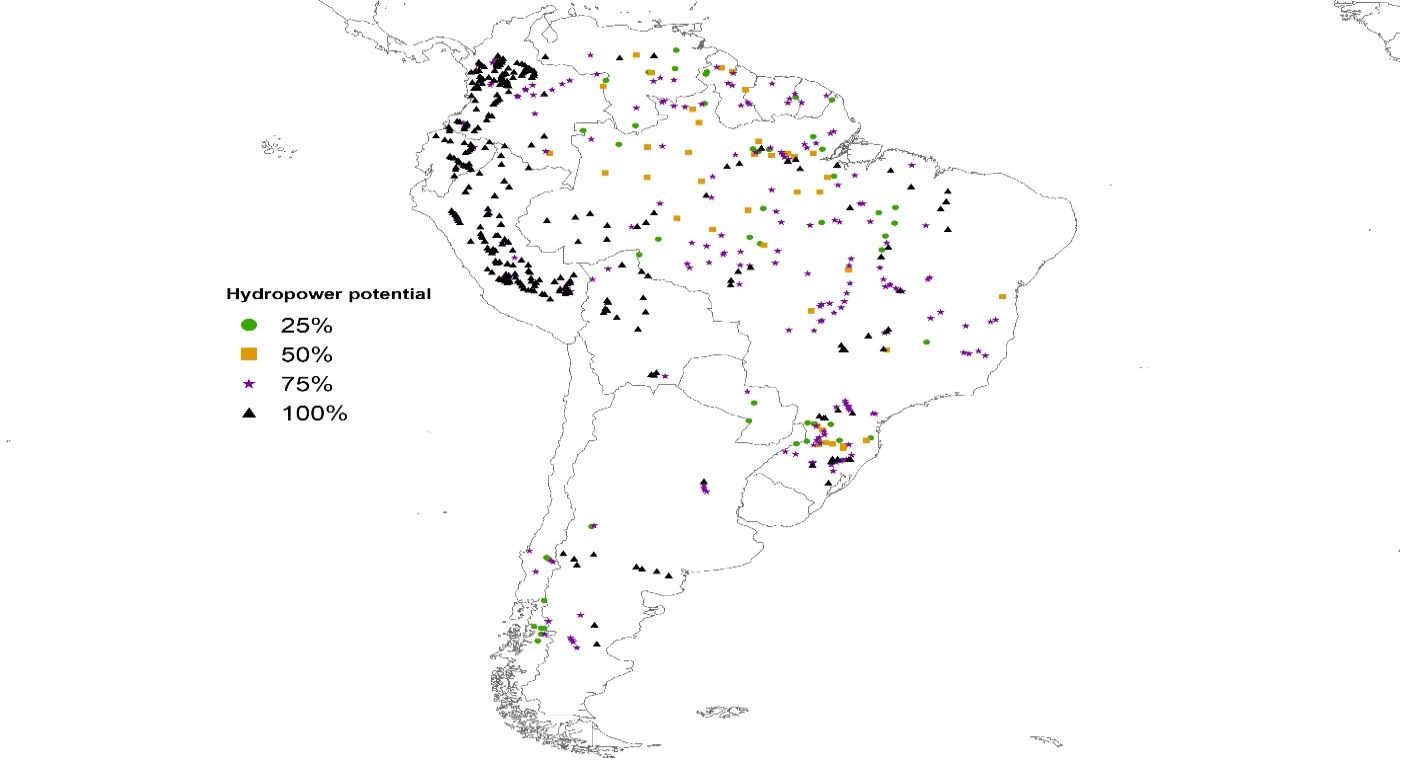


Figure S22: Location of the aquatic biodiversity-impact-wise best hydropower reservoirs, that could be built to reach 25%, 50%, 75% and 100% of the South American hydropower potential. World borders obtained from ref^3^ and created with ArcMap 10.8 (ref^4^).

# S9: Overall biodiversity impact


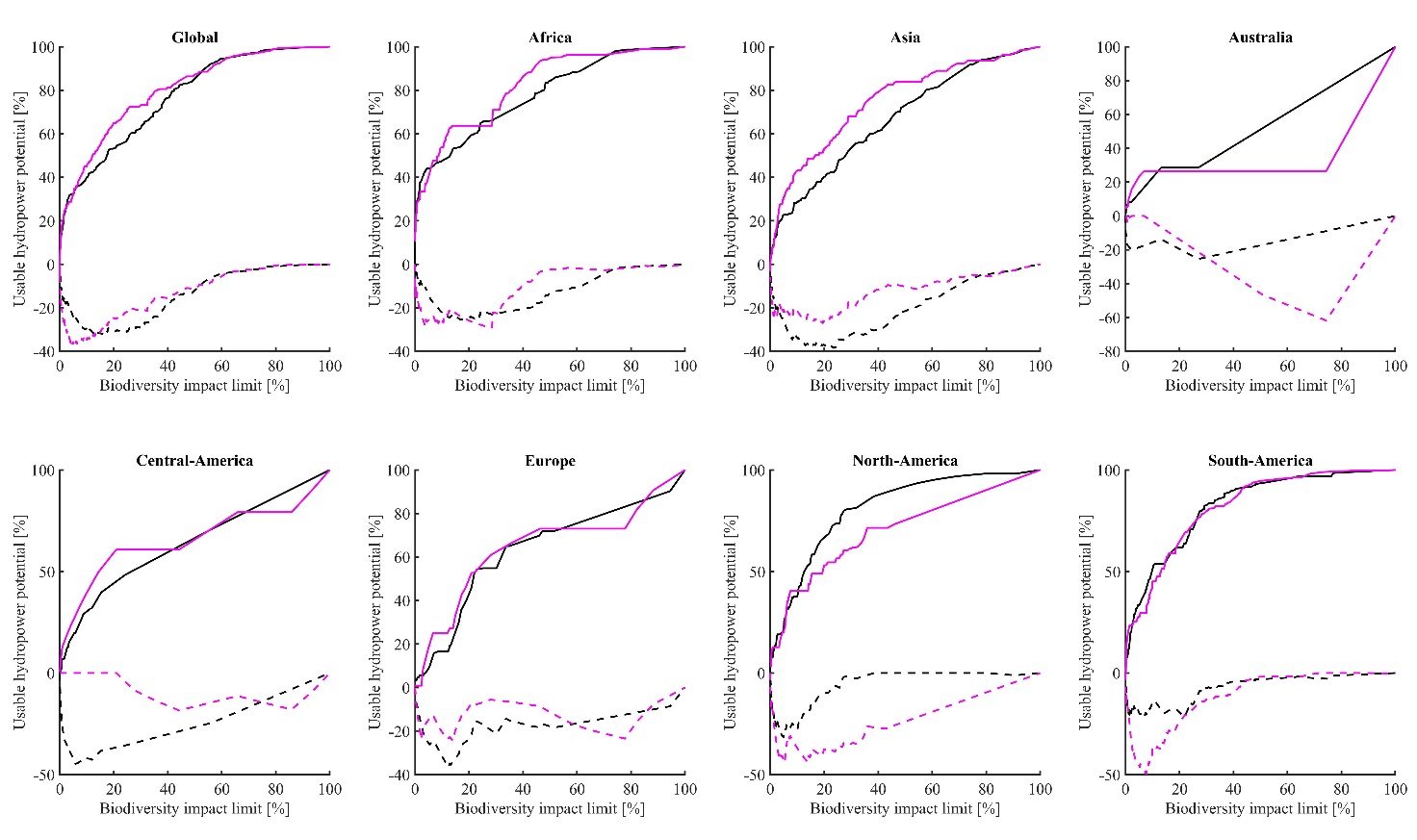


Fig S23: Reduction in % usable hydropower potential (dashed line), and % usable hydropower potential (solid line) on the y-axis for Scenario 1 (black) and 2 (purple), if the biodiversity impact (terrestrial and aquatic) is limited to an equal level (x-axis), in comparison to considering biodiversity impacts (terrestrial and aquatic) individually (dotted line). Scenario 1: reservoirs sorted by terrestrial biodiversity impact and the aquatic biodiversity impacts are considered in parallel. Scenario 2: reservoirs sorted by aquatic biodiversity impact and the terrestrial biodiversity impacts are considered in parallel.

# S10: References used within the supplementary materials

1 Gernaat, D. E. H. J., Bogaart, P. W., Vuuren, D. P. v., Biemans, H. & Niessink, R. High-resolution assessment of global technical and economic hydropower potential. *Nature Energy* **2**, 821-828, doi:10.1038/s41560-017-0006-y (2017).

2 Critical Ecosystem Partnership Fund. *Biodiversity Hotspot Shapefile*, <https://www.cepf.net/our-work/biodiversity-hotspots/hotspots-defined> (2016).

3 Thematic Mapping API. *World Borders Dataset*. http://thematicmapping.org/downloads/world_borders.php>(2009).

4 ESRI. ArcGis Desktop – ArcMap Version 10.8. <https://desktop.arcgis.com/en/arcmap/> (2020).
